# Randomised trial on clinical performances and biocompatibility of four high-flux hemodialyzers in two mode treatments: hemodialysis vs post dilution hemodiafiltration

**DOI:** 10.1038/s41598-019-54404-7

**Published:** 2019-12-04

**Authors:** Marion Morena, Caroline Creput, Mouloud Bouzernidj, Annie Rodriguez, Lotfi Chalabi, Bruno Seigneuric, Céline Lauret, Anne-Sophie Bargnoux, Anne-Marie Dupuy, Jean-Paul Cristol

**Affiliations:** 1 PhyMedExp, Université de Montpellier, INSERM, CNRS, Département de Biochimie et Hormonologie, CHU Montpellier, Montpellier, France; 2 Institut de Recherche et de Formation en Dialyse, Montpellier, France; 3 Service de Néphrologie, AURA, Paris, France; 4 Service de Néphrologie, Clinique Hemera Pays de Caux, Yvetot, France; 5 Département de Biochimie et Hormonologie, CHU Montpellier, Université de Montpellier, Montpellier, France; 6 AIDER, Montpellier, France; 7 Service de Néphrologie, CHU Toulouse, Toulouse, France

**Keywords:** Biochemistry, Haemodialysis

## Abstract

This prospective multicenter randomized comparative cross-over trial aimed at evaluating the influence of hemodialysis vs post-dilution hemodiafiltration with high-flux dialyzers in solute clearance and biocompatibility profile. 32 patients were sequentially dialyzed with Leoceed-21HX, Polypure-22S+, Rexsys-27H and VIE-21A. Primary outcome was β2-microglobulin removal. Secondary outcomes were (i) extraction of other uremic solutes (ii) parameters of inflammation and nutrition and (iii) comparative quantification of perdialytic albumin losses (using total ‘TDC’ vs partial ‘PDC’ collection of dialysate). Significant increases in removal rates of β2-microglobulin (84.7 ± 0.8 vs 71.6 ± 0.8 mg/L), myoglobin (65.9 ± 1.3 vs 38.6 ± 1.3 µg/L), free immunoglobulin light chains Kappa (74.9 ± 0.8 vs 55.6 ± 0.8 mg/L), β-trace protein (54.8 ± 1.3 vs 26.8 ± 1.4 mg/L) and orosomucoid (11.0 ± 1.1 vs 6.0 ± 1.1 g/L) but not myostatin (14.8 ± 1.5 vs 13.0 ± 1.5 ng/mL) were observed in HDF compared to HD when pooling all dialyzers. Rexsys and VIE-A use in both HD and HDF subgroups was associated to a better removal of middle/large-size molecules compared to Leoceed and Polypure, except β2-microglobulin for Rexsys. Inflammatory parameters were unchanged between dialyzers without any interaction with dialysis modality. Mean dialysate albumin loss was comparable between TDC and PDC (1.855 vs 1.826 g/session for TDC and PDC respectively). In addition, a significant difference in albumin loss was observed between dialyzers with the highest value (4.5 g/session) observed using Rexsys. Use of all dialyzers was associated with good removals of the large spectrum of uremic toxins tested and good biocompatibility profiles, with an additional gain in removal performances with HDF. Larger surface area, thinner wall and resultant very high ultrafiltration coefficient of Rexsys should be taken into account in its clear performance advantages.

## Introduction

Ongoing technical improvements in extrarenal therapies are achieved in order to ameliorate the long-term prognosis of chronic kidney disease dialysis patients (CKD-5D)^1,2^. Indeed, efficacy and quality of renal replacement therapy appear among factors affecting patient mortality, the increase in middle molecule clearances ameliorating patient survival in the longer term^3^. Introduction of high-flux membranes first permitted an improvement in dialysis treatment efficiency through enhanced clearances for small and large solutes. Hemodiafiltration (HDF), a convective-based therapy combining both diffusive and convective transports^4^, has been proposed to improve dialysis outcome^5–7^ via a removal spectrum of uremic solutes (small solutes by diffusion and larger molecules with convection)^8^ with an optimized biocompatibility of the extracorporeal circuit obtained with use of ultrapure dialysate and sterile substitution fluids. To date, however, large randomized controlled trials yield conflicting results on its clinical benefits on mortality^9–11^.

Objectives of this study were to evaluate the influence of two dialysis treatments (HD vs post-dilution HDF) with four high-flux (polysulfone or polyethersulfone) dialyzers (with membrane surface area ranging between 2.1 and 2.7 m^2^) (i) in the clearance evaluation for small, middle and large molecular weight substances and (ii) in the modification of several biological responses including parameters of inflammation and nutrition. In addition, a comparative quantification of perdialytic albumin losses using either a partial collection of continuous spent dialysate or the total dialysate collection was performed in a subgroup of patients.

## Results

### Characteristics of the patient population

Between November 2016 and June 2018, 32 CKD-5D patients assessed for eligibility in 4 French dialysis facilities were randomized and followed until their final visit at the latest in July 2018.

Main characteristics of the CKD-5D patients at baseline are listed in Table 1 and dialysis treatment parameters during follow-up are presented in Table 2.

**Table 1:** Baseline characteristics of the studied population.

Parameter	All	HD group	HDF group
N	32	16	16
Gender, Male (%)	20 (62.5%)	9 (56.3%)	11 (68.8%)
Age (years)	72 (10); 73 [42–89]	74 (10); 76 [55–89]	69 (11); 70 [42–84]
Dry weight (kg)	73.0 (17.1); 71.8 [39.5–114.0]	69.9 (13.7); 70.5 [39.5–87.5]	76.1 (19.8); 76.0 [47.5–114.0]
BMI (kg/m^2^)	26.6 (6.9); 25.3 [15.5–49.4]	26.4 (7.7); 23.6 [16.9–49.4]	26.9 (6.2); 27.0 [15.5–36.4]
Hypertension (%)	24 (75.0%)	14 (87.5%)	10 (62.5%)
Diabetes mellitus (%)	10 (31.3%)	6 (37.5%)	4 (25.0%)
Dialysis vintage (years)	4.8 (3.3); 4.4 [0.4–16.6]	5.5 (4.2); 4.7 [0.4–16.6]	4.1 (2.0); 3,6 [1.6–8.6]
Vascular access, AVF (%)	28 (87.5%)	13 (81.3%)	15 (93.8%)
Erythropoiesis stimulating agents (%)	25 (78.1%)	12 (75.0%)	13 (81.3%)
Erythropoiesis stimulating agents (IU/kg/week)	84.8 (60.6); 71.4 [15.7–231.5]	83.2 (64.1); 70.0 [15.7–231.5]	86.3 (59.7); 71.4 [23.6–210.5]
Iron injection (%)	20 (62.5%)	10 (62.5%)	10 (62.5%)
Iron injection (mg/week)	60.0 (35.4); 50.0 [20.0–100.0]	35.0 (25.8); 22.5 [20.0–100.0]	85.0 (24.2); 100.0 [50.0–100.0]
Hemoglobin (g/dL)	11.5 (1.2); 11.5 (8.9–14.2)	11.6 (1.4); 11.6 (8.9–14.2)	11.5 (1.0); 11.4 (9.8–13.7)
Hematocrit (%)	35.4 (3.8); 35.2 (27.0–44.9)	35.7 (4.5); 35.3 (27.0–44.9)	35.0 (3.1); 35.2 (29.9–42.7)
Transthyretin (g/L)	0.31 (0.08); 0.32 (0.14–0.46)	0.29 (0.08); 0.30 (0.14–0.46)	0.32 (0.06); 0.33 (0.22–0.45)
Leukocytes (G/L)	6.5 (2.1); 6.1 (3.8–14.5)	7.2 (2.6); 6.4 (4.3–14.5)	5.8 (1.3); 5.4 (3.8–8.3)
Inorganic phosphates (mmol/L)	1.5 (0.4); 1.4 (0.6–2.3)	1.4 (0.3); 1.4 (0.6–1.9)	1.6 (0.4); 1.6 (0.9–2.3)
C reactive protein (mg/L)	12.4 (22.3); 4.7 (0.6–100.4)	21.1 (29.4); 9.0 (0.8–100.4)	3.7 (2.3); 3.0 (0.6–7.4)

Values were described using proportions for categorical variables and as mean (sd); median [minimum-maximum] for quantitative variables.

**Table 2:** Characteristics of treatment according to the two groups (HD and HDF) during follow-up.

Parameter	Leoceed 21HX				Polypure 22S+				Rexsys 27H				VIE-21A			
	HD		HDF		HD		HDF		HD		HDF		HD		HDF	
	mean	(sd)	mean	(sd)	mean	(sd)	mean	(sd)	mean	(sd)	mean	(sd)	mean	(sd)	mean	(sd)
Duration of dialysis session (h)	3.90	(0.20)	4.00	(0.00)	4.00	(0.10)	3.90	(0.20)	4.00	(0.10)	4.00	(0.10)	3.90	(0.40)	4.00	(0.00)
Blood flow (mL/min)	348.8	(35.8)	374.1	(23.8)	341.3	(35.9)	375.8	(22.9)	346.9	(39.6)	379.4	(23.6)	335.3	(34.2)	370.9	(22.3)
Dialysate flow (mL/min)	561.5	(50.6)	650.0	(51.6)	546.2	(66.0)	650.0	(51.6)	546.2	(66.0)	650.0	(51.6)	546.2	(51.9)	646.7	(51.6)
Intradialytic weight change (kg)	2.5	(3.7)	2.3	(1.0)	1.5	(1.1)	2.2	(1.3)	1.6	(1.2)	2.5	(1.2)	2.3	(1.5)	2.3	(1.2)
Convection volume (L/session)	—	—	23.8	(1.6)	—	—	24.3	(2.0)	—	—	24.6	(1.9)	—	—	22.8	(1.6)

Values were described using mean (sd).

### Dialysis efficacy parameters during the follow-up (see the Methods section for calculations)

- Primary outcome (i.e. performances of b2m removal) is depicted in Table 3.

**Table 3:** Primary outcome: b2m performances with the 4 tested dialyzers using HD and HDF modalities.

Parameter	Leoceed 21HX				Polypure 22 S+				Rexsys 27 H				VIE-21A				ANOVA		
	HD		HDF		HD		HDF		HD		HDF		HD		HDF		Dialyzer	Mode	Dial* Mode
	mean	(sd)	mean	(sd)	mean	(sd)	mean	(sd)	mean	(sd)	mean	(sd)	mean	(sd)	mean	(sd)			
RR (%)	70.9	(1.5)	84.3	(1.5)	67.6	(1.6)	83.5	(1.5)	74.0	(1.6)	85.6	(1.5)	73.9	(1.5)	85.6	(1.6)	0.023	<0.001	NS
RR-eq (%)	57.3	(1.6)	72.1	(1.6)	54.0	(1.8)	70.9	(1.6)	60.5	(1.7)	73.6	(1.6)	59.9	(1.6)	73.6	(1.7)	0.025	<0.001	NS
Kt/V	0.87	(0.05)	1.29	(0.05)	0.79	(0.05)	1.25	(0.05)	0.95	(0.05)	1.34	(0.04)	0.93	(0.05)	1.34	(0.05)	0.026	<0.001	NS
K skm (mL/min)	45.7	(3.4)	74.3	(3.4)	40.8	(3.8)	72.9	(3.4)	50.7	(3.5)	76.3	(3.3)	50.7	(3.4)	76.0	(3.5)	NS	<0.001	NS
Mass (mg)	183.9	(14.9)	216.6	(14.9)	168.6	(16.5)	197.0	(14.9)	186.3	(15.4)	196.4	(14.4)	189.3	(14.9)	197.4	(15.4)	NS	NS	NS
**Parameter**	**Bonferroni post-hoc tests**						**ANOVA Mode**				**ANOVA Dialyzer**								
							**HD**		**HDF**		**Leoceed 21HX**		**Polypure 22S+**		**Rexsys 27H**		**VIE-21A**		
RR (%)	NS						NS		NS		<0.001		<0.001		<0.001		<0.001		
RR-eq (%)	NS						NS		NS		<0.001		<0.001		<0.001		<0.001		
Kt/V	NS						NS		NS		<0.001		<0.001		<0.001		<0.001		
K skm (mL/min)	—						—		—		<0.001		<0.001		<0.001		<0.001		
Mass (mg)	—						—		—		—		—		—		—		

Values were described using mean (sd).

In the ANOVA column, *Dialyzer* means dialyzer effect; *Mode* means mode effect and *Dial* * *Mode* means Interaction between dialyzer and mode.

Bonferroni post-hoc tests correspond to dialyzers effect whatever the mode was. *ANOVA Mode* corresponds to ANOVA testing dialyzers effect according to mode. *ANOVA Dialyzer* corresponds to ANOVA testing mode effects according to dialyzers. (*skm*, simplified kinetic model).
  Regarding *dialysis modality effect*, significantly higher reduction rate (RR) (p < 0.001), Kt/V (p < 0.001) and K simplified kinetic model (skm) (p < 0.001) of serum b2m were observed in HDF compared to HD mode when pooling all dialyzers. These differences observed between modes still persisted within each dialyzer tested. By contrast, b2m mass removal between modes was unchanged.
  Regarding *dialyzer effect*, significantly differences in b2m RR (p = 0.023), equilibrated RR (RR-eq) (p = 0.025) and Kt/V (p = 0.026) were observed between dialyzers whatever the mode was. By contrast, no difference in Kskm and b2m mass removal was reported. In addition, Bonferroni post-hoc tests could not report differences in removal among dialyzers.
- Small solute performances with the tested dialyzers using HD and HDF are presented in Table 4.

**Table 4:** Small solute performances with the 4 tested dialyzers using HD and HDF modalities.

Parameter	Leoceed 21HX				Polypure 22S+				Rexsys 27H				VIE-21A				ANOVA		
	HD		HDF		HD		HDF		HD		HDF		HD		HDF		Dialyzer	Mode	Dial * Mode
	mean	(sd)	mean	(sd)	mean	(sd)	mean	(sd)	mean	(sd)	mean	(sd)	mean	(sd)	mean	(sd)			
(**A**) **Urea** (**60** **Da**)																			
spKt/V	1.80	(0.09)	1.99	(0.08)	1.84	(0.09)	1.94	(0.09)	1.75	(0.10)	1.93	(0.08)	1.90	(0.09)	2.03	(0.09)	NS	0.016	NS
eqKt/V	1.56	(0.07)	1.72	(0.07)	1.59	(0.08)	1.68	(0.07)	1.52	(0.08)	1.66	(0.07)	1.63	(0.07)	1.76	(0.07)	NS	0.012	NS
Kt/V Urea skm	1.33	(0.11)	1.48	(0.11)	1.47	(0.11)	1.59	(0.11)	1.53	(0.12)	1.42	(0.11)	1.40	(0.11)	1.50	(0.11)	NS	NS	NS
RR-eq Urea (%)	73.0	(1.8)	76.5	(1.7)	75.0	(1.7)	76.9	(1.7)	74.7	(1.9)	75.4	(1.7)	74.9	(1.8)	77.1	(1.8)	NS	NS	NS
K skm Urea (mL/min)	209.8	(13.8)	249.0	(13.4)	234.1	(13.4)	265.3	(13.4)	237.6	(14.9)	239.4	(13.4)	224.9	(13.8)	251.1	(13.8)	NS	0.013	NS
Urea mass (mmol)	496.8	(78.3)	583.9	(75.8)	561.8	(78.3)	668.1	(75.8)	513.9	(84.1)	568.3	(75.8)	549.7	(78.3)	564.7	(78.3)	NS	NS	NS
(**B**) **Creatinine** (**113** **Da**)																			
Kt/V Creatinine skm	1.00	(0.06)	1.26	(0.06)	1.00	(0.06)	1.13	(0.06)	1.11	(0.07)	1.12	(0.06)	1.04	(0.07)	1.16	(0.07)	NS	NS	NS
RR-eq Creatinine (%)	62.6	(1.7)	69.6	(1.7)	62.7	(1.7)	66.8	(1.7)	65.9	(1.9)	67.0	(1.7)	64.1	(1.8)	68.1	(1.8)	NS	0.001	NS
K skm Creatinine (mL/min)	158.1	(10.1)	214.2	(10.1)	157.0	(10.5)	193.3	(10.1)	176.8	(11.2)	189.0	(10.1)	166.9	(10.5)	190.1	(10.8)	NS	<0.001	NS
Creatinine mass (µmol × 10^−3^)	15.0	(1.3)	18.3	(1.3)	15.7	(1.3)	18.2	(1.3)	16.2	(1.4)	18.9	(1.3)	15.8	(1.3)	18.0	(1.4)	NS	0.005	NS

Values were described using mean (sd).

**Figure 1:**
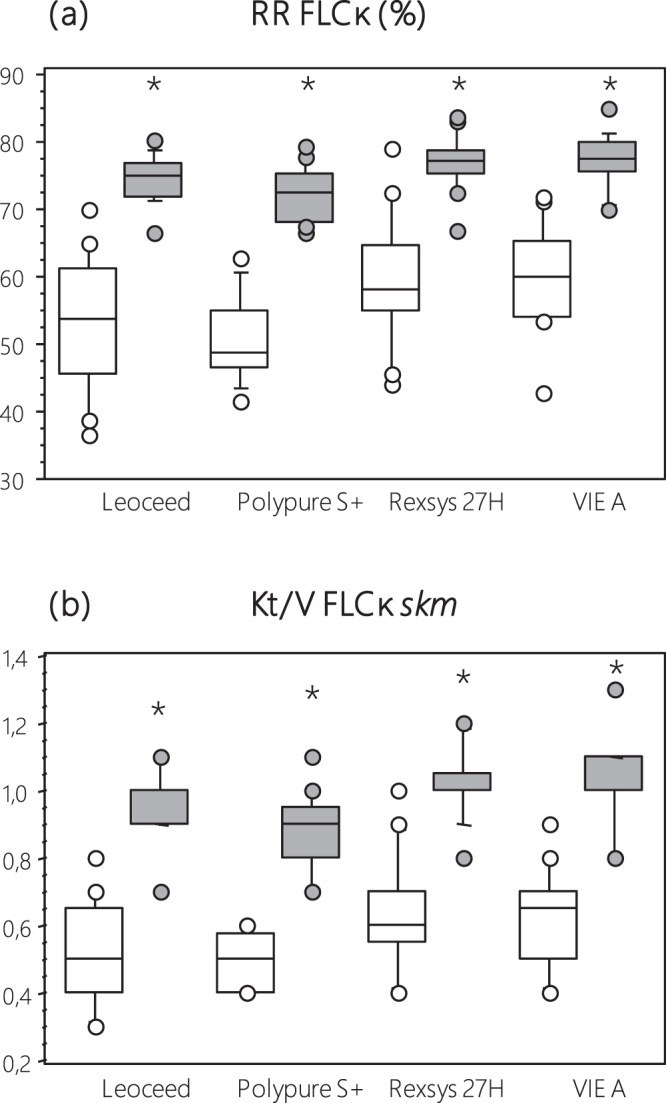
FLC*κ* performances ((**a**) reduction rates RR and (**b**) Kt/V) with the 4 tested dialyzers using HD and HDF modalities. (*FLCκ*, Free Light Chain kappa; RR *reduction ratio; skm*, simplified kinetic model). (□ HD) (■ on line HDF) (*p < 0.001 vs HD mode).

**Figure 2:**
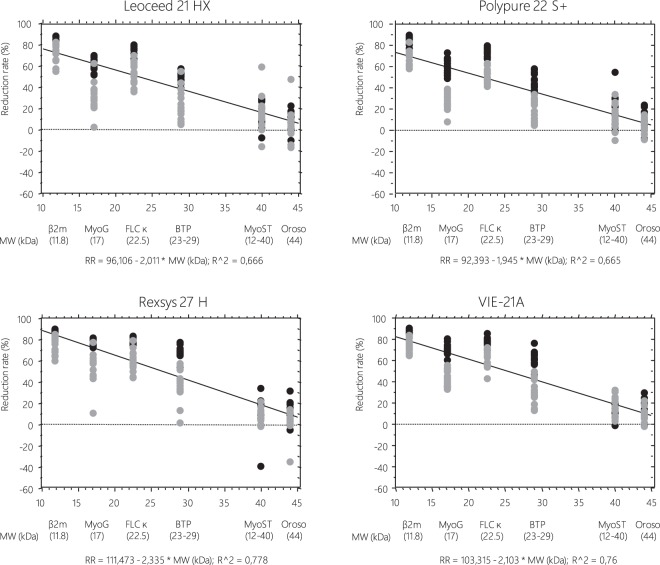
Correlations of the extent of medium molecule removal (reduction rates, %) to their molecular weight with HD or on line HDF technique using the four tested dialyzers. (*β2m*, beta2 microglobulin; *MyoG*, myoglobin; *FLCκ*, Free Light Chain kappa; *BTP*, Beta trace Protein; *MyoST*, myostatin; *Oroso*, orosomucoid). (□ HD) (■ on line HDF).

In the ANOVA column, *Dialyzer* means dialyzer effect; *Mode* means mode effect and *Dial* * *Mode* means Interaction between dialyzer and mode.

(*skm*, simplified kinetic model).
  Regarding urea, HDF mode was associated with a significantly higher single pool spKt/V (p = 0.016), equilibrated eqKt/V (p = 0.012) and effective clearance (p = 0.013) whatever the dialyzer was. By contrast neither effect of dialyzers nor any interaction between dialyzer and mode were observed for these parameters. In addition, no significant difference in urea mass transfer was noted between modes and dialyzers.
  Creatinine RR (p = 0.001), effective clearance (p < 0.001) and mass transfer (p = 0.005) significantly increased in HDF modality without any dialyzer effect.
  Finally, in terms of PO_4_ removal, no significant differences in RR and effective clearance between modes and dialyzers were noted (data not shown).
- Performances of other middle/large-size molecules removal are presented in Table 5, Figs. 1 and 2.

**Table 5:** Other middle and large molecule performances with the 4 tested dialyzers using HD and HDF modalities.

Parameter	Leoceed 21HX				Polypure 22S+				Rexsys 27H				VIE-21A				ANOVA		
	HD		HDF		HD		HDF		HD		HDF		HD		HDF		Dialyzer	Mode	Dial* Mode
	mean	(sd)	mean	(sd)	mean	(sd)	mean	(sd)	mean	(sd)	mean	(sd)	mean	(sd)	mean	(sd)			
**Myoglobin** (**17** **kDa**)																			
RR (%)	31.3	(2.5)	60.5	(2.5)	25.7	(2.6)	59.5	(2.5)	53.3	(2.7)	75.0	(2.5)	44.2	(2.5)	68.5	(2.6)	<0.001	<0.001	NS
RR-eq (%)	22.9	(2.1)	47.5	(2.1)	18.4	(2.2)	46.2	(2.1)	41.1	(2.3)	61.4	(2.1)	32.6	(2.1)	55.1	(2.2)	<0.001	<0.001	NS
Kt/V	0.27	(0.04)	0.66	(0.04)	0.21	(0.04)	0.63	(0.04)	0.55	(0.04)	0.96	(0.04)	0.40	(0.04)	0.82	(0.04)	<0.001	<0.001	NS
**FLCκ** (**22**.**5** **kDa**)																			
RR (%)	53.3	(1.7)	73.8	(1.7)	50.4	(1.7)	72.2	(1.7)	58.8	(1.7)	76.2	(1.6)	59.7	(1.7)	77.4	(1.7)	<0.001	<0.001	NS
RR-eq (%)	40.8	(1.6)	60.3	(1.6)	38.2	(1.6)	58.3	(1.6)	45.7	(1.6)	62.7	(1.5)	46.1	(1.6)	64.1	(1.6)	<0.001	<0.001	NS
Kt/V	0.53	(0.03)	0.93	(0.03)	0.48	(0.03)	0.88	(0.03)	0.62	(0.03)	1.00	(0.03)	0.63	(0.03)	1.03	(0.03)	<0.001	<0.001	NS
**BTP** (**23–29** **kDa**)																			
RR (%)	22.4	(2.7)	47.3	(2.7)	19.3	(2.8)	45.0	(2.7)	35.2	(2.8)	67.7	(2.6)	30.2	(2.7)	59.1	(2.8)	<0.001	<0.001	NS
RR-eq (%)	16.2	(2.1)	35.7	(2.1)	13.8	(2.2)	33.7	(2.1)	26.0	(2.2)	54.0	(2.1)	21.7	(2.1)	46.2	(2.2)	<0.001	<0.001	NS
Kt/V	0.19	(0.03)	0.45	(0.03)	0.15	(0.03)	0.42	(0.03)	0.31	(0.03)	0.78	(0.03)	0.25	(0.03)	0.63	(0.03)	<0.001	<0.001	NS
**Myostatin** (**12–40** **kDa**)																			
RR (%)	14.8	(3.0)	15.0	(3.0)	11.9	(3.1)	17.4	(3.0)	7.6	(3.1)	11.9	(2.9)	17.6	(3.0)	14.8	(3.1)	NS	NS	NS
RR-eq (%)	10.7	(2.1)	10.7	(2.1)	8.5	(2.2)	12.5	(2.1)	5.3	(2.2)	8.5	(2.1)	12.4	(2.1)	10.5	(2.2)	NS	NS	NS
Kt/V	0.12	(0.03)	0.12	(0.03)	0.09	(0.03)	0.14	(0.03)	0.06	(0.03)	0.10	(0.02)	0.14	(0.03)	0.11	(0.03)	NS	NS	NS
**Orosomucoid** (**44** **kDa**)																			
RR (%)	5.4	(2.1)	10.6	(2.1)	3.8	(2.3)	9.2	(2.1)	6.8	(2.2)	11.4	(2.1)	7.9	(2.1)	12.6	(2.2)	NS	0.002	NS
RR-eq (%)	3.9	(1.5)	7.5	(1.5)	2.7	(1.6)	6.5	(1.5)	4.7	(1.6)	8.1	(1.5)	5.5	(1.5)	8.9	(1.6)	NS	0.002	NS
Kt/V	0.05	(0.02)	0.08	(0.02)	0.03	(0.02)	0.07	(0.02)	0.05	(0.02)	0.09	(0.02)	0.06	(0.02)	0.10	(0.02)	NS	0.003	NS
**Parameter**	**Bonferroni post-hoc tests**						**ANOVA Mode**				**ANOVA Dialyzer**								
							**HD**		**HDF**		**Leoceed 21HX**		**Polypure 22S+**		**Rexsys 27H**		**VIE-21A**		
**Myoglobin** (**17** **kDa**)																			
RR (%)	Rexsys, VIE-A > Leoceed, Polypure						<0.001		<0.001		<0.001		<0.001		<0.001		<0.001		
RR-eq (%)	Rexsys > VIE-A > Leoceed, Polypure						<0.001		<0.001		<0.001		<0.001		<0.001		<0.001		
Kt/V	Rexsys > VIE-A > Leoceed, Polypure						<0.001		<0.001		<0.001		<0.001		<0.001		<0.001		
**FLCκ** (**22**.**5** **kDa**)																			
RR (%)	Rexsys, VIE-A > Leoceed, Polypure						0.008		0.006		<0.001		<0.001		<0.001		<0.001		
RR-eq (%)	Rexsys, VIE-A > Leoceed, Polypure						0.011		0.005		<0.001		<0.001		<0.001		<0.001		
Kt/V	Rexsys, VIE-A > Leoceed, Polypure						0.011		0.004		<0.001		<0.001		<0.001		<0.001		
**BTP** (**23**–**29** **kDa**)																			
RR (%)	Rexsys > VIE-A > Leoceed, Polypure						0.003		<0.001		<0.001		<0.001		<0.001		<0.001		
RR-eq (%)	Rexsys > VIE-A > Leoceed, Polypure						0.003		<0.001		<0.001		<0.001		<0.001		<0.001		
Kt/V	Rexsys > VIE-A > Leoceed, Polypure						0.004		<0.001		<0.001		<0.001		<0.001		<0.001		
**Myostatin** (**12**–**40** **kDa**)																			
RR (%)	—						—		—		—		—		—		—		
RR-eq (%)	—						—		—		—		—		—		—		
Kt/V	—						—		—		—		—		—		—		
**Orosomucoid** (**44** **kDa**)																			
RR (%)	—						—		—		NS		NS		NS		NS		
RR-eq (%)	—						—		—		NS		NS		NS		NS		
Kt/V	—						—		—		NS		NS		NS		NS		

Values were described using mean (sd).

In the ANOVA column, *Dialyzer* means dialyzer effect; *Mode* means mode effect and Dial * Mode means Interaction between dialyzer and mode.

Bonferroni post-hoc tests correspond to dialyzers effect whatever the mode was. *ANOVA Mode* corresponds to ANOVA testing dialyzers effect according to mode. *ANOVA Dialyzer* corresponds to ANOVA testing mode effects according to dialyzers. (*BTP*, β Trace Protein; *FLCκ*, Free Light Chain kappa).

Regarding *dialysis modality effect*, significantly higher RR, RR-eq and Kt/V of myoG (p < 0.001), FLCκ (p < 0.001), BTP (p < 0.001) and orosomucoid (p = 0.002) were observed in HDF compared to HD mode whatever the dialyzer was. These differences observed between modes still persist within each dialyzer tested except for orosomucoid. Finally, no significant difference in myoS removal performances was reported between modes.

Regarding *dialyzer effect*, significantly differences in RR, RR-eq and Kt/V of serum myoG, FLCκ and BTP but not myoS and orosomucoid were observed between dialyzers whatever the mode was. In addition, Bonferroni post-hoc tests reported that Rexsys use was associated to a better removal of middle/large-size molecules except myoS and orosomucoid compared to Leoceed and Polypure. Its dialysis performances were comparable or even superior to VIE-A ones depending on the parameter tested: comparable for FLCk (RR, RR-eq and Kt/V) and myoG (RR); superior for BTP (RR, RR-eq and Kt/V) and myoG (RR-eq and Kt/V).

### Biocompatibility parameters during the follow-up

Impact of tested dialyzers on bioincompatibility parameters using HD and HDF modalities is reported in Table 6. No significant change in pre-dialysis levels of albumin and transthyretin was observed between HD and HDF groups and between all dialyzers. By contrast, pre-dialysis levels of CRP and orosomucoid were significantly lower in HDF with use of Leoceed (p = 0.042 for CRP only), Polypure (p = 0.049 and 0.026 for CRP and orosomucoid respectively), Rexsys (p = 0.004 for orosomucoid only) and VIE-A (p = 0.017 for orosomucoid only).

**Table 6:** Biocompatibility parameters with the 4 tested dialyzers using HD and HDF modalities.

Parameter	Leoceed 21HX				Polypure 22S+				Rexsys 27H				VIE-21A				ANOVA		
	HD		HDF		HD		HDF		HD		HDF		HD		HDF		Dialyzer	Mode	Dial* Mode
	mean	(sd)	mean	(sd)	mean	(sd)	mean	(sd)	mean	(sd)	mean	(sd)	mean	(sd)	mean	(sd)			
Albumin (g/L)	36.1	(0.8)	38.5	(0.8)	38.0	(0.8)	37.6	(0.8)	37.1	(0.8)	37.8	(0.8)	36.2	(0.8)	37.9	(0.8)	NS	NS	NS
Transthyretin (g/L)	0.298	(0.020)	0.321	(0.020)	0.310	(0.020)	0.314	(0.020)	0.333	(0.021)	0.315	(0.020)	0.307	(0.020)	0.320	(0.021)	NS	NS	NS
CRP (mg/L)	23.7	(8.6)	6.8	(8.6)	11.2	(8.6)	4.2	(8.6)	11.4	(8.9)	3.6	(8.3)	29.9	(8.6)	11.5	(8.9)	NS	0.042	NS
Orosomucoid (g/L)	1.37	(0.09)	1.05	(0.09)	1.20	(0.09)	0.98	(0.09)	1.24	(0.09)	0.96	(0.09)	1.36	(0.09)	0.98	(0.09)	NS	<0.001	NS
IL-6 (Pre) (pg/mL)	9.8	(2.0)	5.5	(2.0)	4.3	(2.0)	3.9	(2.0)	5.1	(2.0)	3.6	(1.9)	9.4	(2.0)	5.4	(2.0)	NS	NS	NS
IL-6 (Post) (pg/mL)	9.9	(1.8)	6.2	(1.8)	4.6	(1.8)	3.7	(1.8)	3.6	(1.9)	3.1	(1.7)	7.4	(1.8)	4.3	(1.8)	NS	NS	NS
TNF-α (Pre) (pg/mL)	29.3	(1.9)	29.5	(1.9)	28.6	(1.9)	27.6	(1.9)	28.5	(2.0)	25.3	(1.9)	28.9	(1.9)	27.9	(2.0)	NS	NS	NS
TNF-α (Post) (pg/mL)	13.0	(1.3)	11.9	(1.3)	12.9	(1.4)	11.8	(1.3)	13.0	(1.4)	11.0	(1.3)	14.0	(1.4)	10.6	(1.4)	NS	NS	NS

Values were described using mean (sd).

In the ANOVA column, *Dialyzer* means dialyzer effect; *Mode* means mode effect and *Dial* * *Mode* means Interaction between dialyzer and mode.

Regarding IL-6 and TNF-α, both pre- and post-dialysis levels remained unchanged over the study period whatever the modality or the dialyzer was.

### Albumin loss with use of the four tested dialyzers

**Figure 3:**
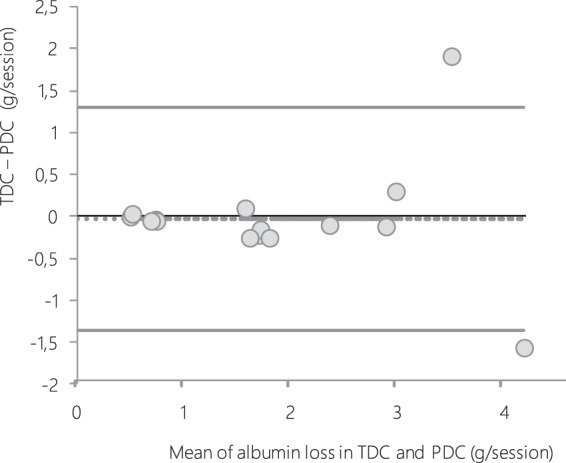
Bland & Altman bias plot for albumin loss measured from direct dialysis quantification using total (TDC) and partial (PDC) spent dialysate collection. For technical reasons, this comparative quantification was evaluated in only half of the patients from center 1 (n = 4). Dotted line represents the mean difference and solid lines represent 95% limits of agreement. All values are expressed in g/session.

*The comparative quantification* (*PDC versus TDC*) *of albumin loss*, based on 16 comparisons reckoning concurrent PDC and TDC in 4 patients dialysed sequentially with the four different dialyzers, is depicted in the Bland–Altman bias analysis (Fig. 3). Limits of agreement with 95% confidence intervals (CI) were −1,36 to 1,30. The deviation of dialysate albumin loss estimated using TDC vs PDC is minimal with no systematic bias. Despite the low number of subjects, the agreement may be considered as acceptable.

Mean values of dialysate albumin removal were 1.855 and 1.826 g/session for “TDC” and “PDC” respectively.

**Figure 4:**
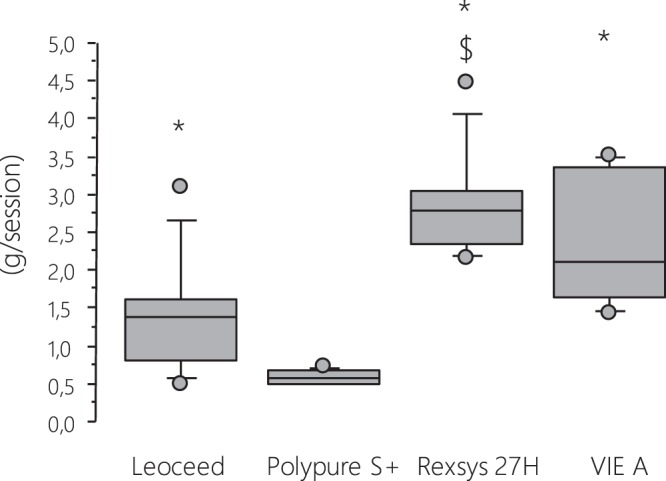
Dialysate loss of albumin in post-dilution HDF with the 4 tested dialyzers. Continuous partial sampling of spent dialysate with a collection pump inserted into the dialysate outlet line was carried out in all patients from center 1 during the midweek session of each four treatment phases. At the end of the session, a sample was collected after stirring and albumin determined. (*p < 0.05 vs Polypure S+; ^$^p < 0.05 vs Leoceed).

*The comparative analysis of albumin loss with the four tested dialyzers using PDC method* is presented in Fig. 4. Dialysate loss of albumin with post-dilution HDF was significantly lower with Polypure compared to Leoceed (p = 0.0173), Rexsys (p = 0.0117) and VIE-A (p = 0.0180). In addition, a significant difference was observed between Leoceed and Rexsys (p = 0.0251). Overall, total albumin mass was slightly removed with Polypure [median 0.6 (range 0.5–0.7) g] and moderately removed with Leoceed [median 1.4 (range 0.5–3.1) g], VIE-A [median 2.1 (range 1.4–3.5) g] and Rexsys [median 2.8 (range 2.1–4.5) g].

## Discussion

CKD-5D patient survival in the long term may be improved by increasing efficacy and quality of renal replacement therapy. Among factors, treatment modality and types of dialyzer used may have a strong impact on clinical outcomes^12^. Purpose of this prospective observational study thus aimed at evaluating the detailed characterization of efficacy and biocompatibility performances of four dialyzers using HD and HDF modalities. Efficacy evaluation was based on a set of small, middle- and large-sized molecules in the range 11.8–44 kDa. Our results confirmed the experienced superiority of HDF over HD in middle/large-sized molecule removal including b2m, myoG, FLCκ, BTP and orosomucoid whatever the dialyzer was. Dialysis performances of Rexsys were higher compared to Leoceed and Polypure but comparable or slightly above VIE-A ones depending on the tested parameters. Inflammatory parameters were unchanged between dialyzers without any interaction with dialysis modality. Dialysate albumin content in HDF was comparable using both dialysate collections. Significant differences in perdialytic losses were noted between dialyzers but the overall leaking was estimated <4.5 g/session.

Only subtle differences in removal were observed for small solutes with respect to dialysis modality, with slightly but significantly increased removal of urea and creatinine with HDF. The eqKt/V in the range 1.52–1.63 in HD and 1.66–1.76 in HDF observed here were expected since removal of small solutes mainly occurs via diffusion, the additional convection in HDF modality not affecting diffusive transport. It is generally accepted that HDF is more efficient in the removal of larger molecules. However, our results corroborate those from previous studies reporting a moderate increase in small molecules removal with HDF modality^13,14^.

Regarding middle/larger-sized molecules, as expected, HDF was associated to improved removal performances with all tested dialyzers and among dialyzers, Rexsys and VIE-A were the most efficient ones especially in myoG, FLCκ and BTP removal. These differences observed may be attributable to the higher effective surface of Rexsys, mainly its high cut-off, and the narrowed inner diameter of fibers for VIE-A which allows to optimize internal convective transport thus increasing clearance of middle/large size molecules. In HD mode, myoG RR obtained with VIE-A were higher than those reported with FX100 and close to FXCordiax-100 as previously reported^15^. In the same manner, we observed excellent myoG RR with Rexsys in HD, comparable to those reported with FXCordiax-100^15,16^. This may be due to the higher membrane surface of Rexsys (2.7 m^2^ vs 2.2 m^2^ for FX-Cordiax100) and the narrowed inner diameter of fibers for FXCordiax-100 (185 µm vs 200 µm for Rexsys). Even though HDF could improve b2m removal performances, no difference between dialyzers was observed neither in HD nor in HDF, with b2m RR close to those found with FX80^17^, FXCordiax-80^17,18^ and FXCordiax-100^15,16^ in HD and higher than FXCordia-100 in HDF^15^.

Excellent RR were observed for FLCκ removal with levels greater than 70% in HDF, even reaching 76% with Rexsys, which is higher than those previously observed with Elisio™-210H, a dialyzer whose nature of the Polynephron™ membrane and the design of the dialyzer, mainly its effective longer length, allow huge internal convection^19^. Of note, despite a higher molecular weight, FLCκ RR reported here are higher than myoG ones.

Regarding other large size molecules studied here, only few studies explored the removal of oromucoid and myoS. Here, we noted a difference in orosomucoid RR between modalities but without any dialyzer effect. These levels observed in HDF represent around 10–12%. Maduell *et al*. using FXCordiax-60, with lower membrane surface (1.4 m^2^) but narrowed inner diameter of fibers (185 µm) could reach similar rates but with longer time dialysis sessions^20^. In terms of myoS, our findings could evidence removals up to 17.6% but without significant differences between modalities and between dialyzers. ELISA kit used here allows the determination of the active mature protein of myoS which can be found in the circulation either as a 12 kDa fragment (free mature protein) (25% in the circulation) or a 40 kDa fragment (75%) corresponding to a latent complex containing a disulfide-linked dimer of the mature protein and two noncovalently-associated propeptides. This should explain why only weak RR were observed here, probably attributable to the predominant 40 kDa form of myoS present in the circulation. Interestingly, Esposito *et al*., using the same ELISA kit (R&D Systems) could report decreased pre-dialysis levels of myoS after moving from a 3 month-HD period to a 3 month-HDF period but in only 10 patients^21^.

Our findings validate the partial dialysate collection by direct dialysate quantification of perdialytic albumin losses. This PDC appears as a reliable technique reflecting albumin losses in TDC and may be considered as a suitable method to determine total dialysate content of any solute. In our study, it must be underlined that along with their efficacy performances on middle/large-sized molecule removal, all tested dialyzers did not cause dramatic albumin leaking with HDF treatment. Indeed, even though we noted significant differences between dialyzers, with a higher amount of albumin leaking using Rexsys, the mean level did not exceed 2.8 g/3–4 hour-treatment, which corresponds to acceptable amounts in HDF compared to other studies^22,23^. These levels are even lower than some reported with HD, as observed by Kirsch *et al*. with up to 7.3 g/session of albumin leaking using middle cut-off membranes^18^. Nevertheless, the amount of albumin loss during dialysis that is clinically acceptable in the long term still needs investigations all the more so because the main cause of low serum albumin levels in hypoalbuminemic dialysis patients is decreased albumin synthesis rather than perdialytic losses^24,25^.

Lack of cellular reactivity with inflammatory parameters observed with all dialyzers attests to their high biocompatibility level. Indeed, It is noteworthy that all the tested parameters were not modified during the sessions performed with both techniques which clearly shows the excellent biocompatibility profile achieved with today’s dialysis procedure^26^, all the more with HDF wherein high volumes of reinfusion liquid are performed^27^.

The major limitation of this study is the acute conditions of the design which not allowed to address whether use of these dialyzers in HDF mode was superior to HD in terms of clinical outcomes. In addition, before enrolment, the patients were treated with HDF or HD and no run-in was planned. This may explain the differences observed between study populations at inclusion and during the study in terms of inflammatory markers. Given the short term of the study, therefore a carry-over effect cannot be ruled out. Finally, patients in the HDF group had significantly higher blood and dialysate flow rates which let suggest increased treatment performances with this mode. However, instantaneous clearance curve of small solute and larger molecules reaches a steady state around 350 mL/min for blood flow and 500 mL/min for dialysate flow both in HD and HDF modalities^28^. Therefore, only a moderate effect could be attributed to this difference.

In conclusion, use of the different tested dialyzers was associated with a good removal of the large spectrum of uremic toxins tested (higher performances being obtained with Rexsys and VIE-A dialyzers) and an excellent biocompatibility profile allowing their safe use for both modalities. Larger surface area, thinner wall and resultant very high ultrafiltration coefficient of Rexsys should be taken into account in its clear performance advantages.

## Methods

### Ethical approval

All procedures performed in this study involving human participants were in accordance with the ethical standards of the institutional and/or national research committee and with the 1964 Helsinki declaration and its later amendments or comparable ethical standards.

### Study design, dialyzers and dialysis conditions

**Figure 5:**
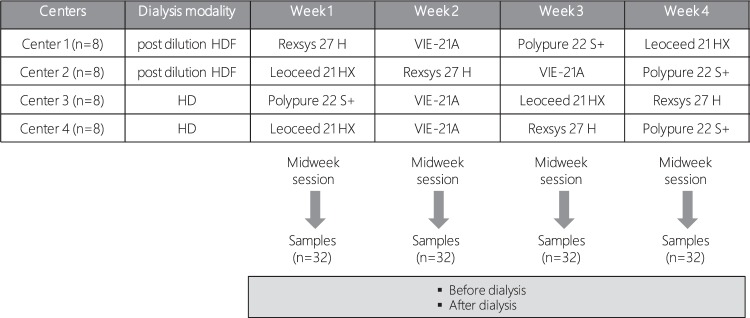
Study design and sequences of randomization per center. In this prospective multicenter randomized comparative cross-over trial in two parallel arms, HD vs on line HDF, all the patients were sequentially dialyzed with four different dialyzers (one/week): Leoceed 21HX, Polypure 22S+, Rexsys 27H and VIE-21A.

This study was a prospective multicenter randomized comparative cross-over trial (ClinicalTrials.gov Identifier: NCT03262272; first posted 25/08/2017), registered at “Ministère Santé et Solidarités” after approval by the Marseille University Hospital ethics committee. Four different dialysis facilities (AURA Paris, Yvetot clinic, AIDER clinic and Toulouse university hospital center) were involved in the study. Eligible patients were assigned to receive either post-dilution HDF (first two centers) or conventional high-flux HD (last two centers) for 4 weeks. During this period, all patients were sequentially dialyzed with 4 different dialyzers (one/week, the two first sessions of the week; the last session of the week being performed with the patient’s own dialyzer and used as a wash out session): Leoceed 21HX, Polypure 22S+, Rexsys 27H and VIE-21A (Table 7). The sequence of dialyzers use was randomly assigned: per center, device × week allocation scheme was generated by random permutation of a block of 4 (computer generation). All patients in a given center followed the scheme allocated to this center. (Fig. 5). The randomization sequence was centralized and computed by the statistician.

**Table 7:** Characteristics of the membranes in the tested dialyzers.

	Membrane	Effective surface area (m^2^)	Inner diameter (µm)	Wall thickness (µm)	UF coefficient (mL/h/mm Hg)
Leoceed 21HX	polysulfone	2.1	200	35	94
Polypure 22S+	polysulfone	2.2	200	40	74
Rexsys 27H	polyethersulfone	2.7	200	30	124
VIE-21A	vitamin E-coated polysulfone	2.1	185	45	89

Dialysis conditions remained unchanged for each patient: 3 sessions/week, 3–4 hours/session, blood flow (QB) of 300–400 mL/min, ultrapure bicarbonate buffered dialysate, dialysate flow (QD) in the range 500–700 mL/min. On-line production of clean dialysate and ultrapure substitution fluid was performed in both modalities. HDF was performed in post-dilution mode with a minimum convective volume of 20 L^29,30^. The ultrafiltration flow rate (QUF) was set according to each individual patient’s interdialytic weight gain. The routine anticoagulation protocol was unchanged for each patient.

Primary outcome measure of the study was the removal of β2-microglobulin (b2m). Secondary outcome measures were (i) the extraction of other uremic solutes including myoglobin (myoG), free immunoglobulin light chains Kappa (FLCκ), β-trace protein (BTP), myostatin (myoS), glycoprotein orosomucoid, urea, creatinine and phosphates (PO_4_), (ii) parameters of inflammation (CRP,TNF-α,IL-6), nutrition (albumin, transthyretin) and (iii) a comparative quantification of perdialytic albumin losses.

Informed consent was obtained from all individual participants.

### Patients

Thirty-two hemodialysis patients (n = 8 per center) were enrolled in the study by their nephrologists after having fulfilled the following inclusion criteria: adult patient with CKD, dialyzed for at least one month with high permeability and high surface area (≥1,8 m^2^) dialyzer and treated with the mode to be tested (HD or post-dilution HDF); with a minimum convective volume of 20 L in post-dilution HDF; with vascular access allowing a minimum blood flow rate of 300 mL/min; informed of the study goals and having signed the written informed consent.

Non inclusion criteria were as follows: patient with a fast progressive acute/chronic disease; with uncontrolled anemia; pregnant or nursing patient.

No run-in period before entering in the active study phase was performed.

### Blood sample collection and laboratory measurements

Blood samples were drawn weekly during the midweek dialysis session from the arterial line before and after dialysis.

Routine laboratory analyses including serum urea, creatinine and PO_4_ (all being evaluated pre- and post-dialysis) were locally performed. Specific biomarkers including serum b2m, myoG, FLCκ, BTP, myoS, orosomucoid, CRP, TNF-α, IL-6, albumin, transthyretin were performed on samples collected weekly, centrifuged, aliquoted, frozen and further analyzed in a central laboratory.

Serum b2m, orosomucoid, high sensitive CRP, albumin and transthyretin were determined by immunoturbidimetry (Cobas 8000, Roche Diagnostics, Meylan, France). MyoG was measured by electrochemiluminescence immunoassay (Cobas 8000, Roche Diagnostics, Meylan, France). FLCκ and BTP were evaluated using latex-enhanced immunonephelometry (Siemens Healthcare Diagnostics Products, Marburg, Germany). MyoS, TNF-α and IL-6 were measured by enzyme-linked immunosorbent assay (myoS: R&D Systems Europe, Lille, France; TNF-α/IL-6: ThermoFisher Scientific, Courtaboeuf, France).

### Dialysate sample collection and albumin measurement

A comparative quantification of perdialytic albumin losses using either a “pull/push” syringe allowing to collect continuous spent sampling of dialysate (namely partial dialysate collection, “PDC”) or the total dialysate collection (namely “TDC”), which represents the gold standard method to evaluate mass balances achieved during dialysis for a given solute^31^, was performed in a subgroup of patients (i.e. center-1), all being dialyzed with post-dilution HDF (n = 8 patients for PDC and n = 4 for TDC due to the cumbersome method).

Briefly, total amount of spent dialysate with ultrafiltrate (TDC) was collected in a 200 L tank during the midweek session of each four treatment phases. At the end of the session, a 60 mL-sample was collected after a manual 8-minute stirring and sent to the laboratory for albumin determination. Regarding PDC, a continuous partial sampling of spent dialysate was carried out using a collection pump inserted into the dialysate outlet line (waste dialysate) at a sampling rate of 14.5 mL/hour for a total volume of 48 mL. The sampler was annexed to hydraulic alarms of the generator in order to avoid fresh dialysate inlet. At the end of the session, the total volume of the syringe was sent to the laboratory for albumin determination. Albumin dialysate content for TDC and PDC was assayed by immunoturbidimetric method using Beckman-Coulter AU5800 analyzer (Villepinte, France).

### Calculations

- *Dialysis dose delivered for urea* was calculated using single pool (sp)^32^ and equilibrated (eq)^33^ Kt/V according to following Daugirdas formulas:
  $$\begin{array}{rcl}{\rm{sp}}\,\mathrm{Kt}/V & = & -\,\mathrm{Ln}\,([{{\rm{urea}}}_{{\rm{post}}}]/[{{\rm{urea}}}_{{\rm{pre}}}])-0.008\,\ast \,{\rm{tHD}}\\  &  & +\,((4-3.5\,\ast \,[{{\rm{urea}}}_{{\rm{post}}}]/[{{\rm{urea}}}_{{\rm{pre}}}])({{\rm{BW}}}_{{\rm{pre}}}-{{\rm{BW}}}_{{\rm{post}}})/{{\rm{BW}}}_{{\rm{post}}}))\end{array}$$
  $${\rm{eq}}\,\mathrm{Kt}/V={\rm{sp}}\,\mathrm{Kt}/V-(0.6\,\ast \,{\rm{sp}}\,\mathrm{Kt}/V/{\rm{tHD}})+0.03$$
  where *sp* is single pool, *pre* and *post* correspond to pre- and post-treatment conditions respectively, tHD is time on dialysis in hours, [urea] is urea concentration in mmol/L and BW is Body Weight in kg.
- *Reduction rates* of different solutes were calculated according to the following equation:
  $${\rm{RR}}\,( \% )=({{\rm{C}}}_{{\rm{pre}}}-{{\rm{C}}}_{{\rm{post}}})/{{\rm{C}}}_{{\rm{pre}}}\,\ast \,100$$
  where C_pre_ and C_post_ are solute concentrations pre- and post-treatment respectively.
  For the middle- and large-size molecules, C_post_ was corrected (cpost-corr) for hemoconcentration, using a single compartment kinetic model^34^:
  $${{\rm{C}}}_{\mathrm{post}-\mathrm{corr}}={{\rm{C}}}_{{\rm{post}}}/(1+\Delta \mathrm{BW}/(0,2\,\ast \,{\rm{BW}}\,{\rm{post}}))$$
  where ∆BW is the difference between pre and post-treatment body weights (BW).
- *Equilibrated reduction rates* of different solutes were calculated according to the following equation:
  $$\mathrm{RR}-\mathrm{eq}\,( \% )=({{\rm{C}}}_{{\rm{pre}}}-{{\rm{C}}}_{\mathrm{post}-\mathrm{eq}})/{{\rm{C}}}_{{\rm{pre}}}\,\ast \,100$$
  where C_post-eq_ corresponds to post-treatment concentration corrected for solute compartment disequilibrium according to Tattersall formula^35^:
  $${{\rm{C}}}_{\mathrm{post}-\mathrm{eq}}={{\rm{C}}}_{{\rm{pre}}}\,\ast \,{({{\rm{C}}}_{{\rm{post}}}/{{\rm{C}}}_{{\rm{pre}}})}^{({\rm{tHD}}/{\rm{tHD}}+{\rm{X}})}$$
  where X corresponds to the equilibration time and equals to 35 and 50 for urea and creatinine respectively.
  For the middle- and large size molecules, the following formula considering an equilibration time of 110 was applied^36^.
- *Kt/V for all solutes* was calculated using simplified kinetic model (skm) according to the following equation:
  $${\rm{Kt}}/{\rm{V}}\,{\rm{solute}}=\,\mathrm{Ln}({{\rm{C}}}_{{\rm{pre}}}/{{\rm{C}}}_{\mathrm{post}-\mathrm{eq}})$$
- *Effective clearances for solutes* were calculated according to the following equation:
  $${\rm{K}}\,{\rm{skm}}\,{\rm{solute}}=\,\mathrm{Ln}({{\rm{C}}}_{{\rm{pre}}}/{{\rm{C}}}_{\mathrm{post}-\mathrm{eq}})\,\ast \,{\rm{V}}/{\rm{tHD}}$$
  where V corresponds to the volume of distribution for solutes and was calculated using the Chertow total body water (TBW) formula^37^ as follows:
  V = averaged Total Body Water (TBW) for urea and creatinine
  V = One third of averaged TBW for middle- and large-size molecules
  with averaged TBW = (TBW + (TBW-∆BW))/2
- *Solute mass removal* during the sessions was estimated using the following formula:
  $${\rm{Mass}}\,{\rm{solute}}={\rm{K}}\,{\rm{solute}}\,\ast \,0,001\,\ast \,{\rm{TAC}}\,{\rm{solute}}\,\ast \,{\rm{tHD}}$$
  where TAC is time average concentration of the solute and equals to:
  $${\rm{TAC}}\,{\rm{solute}}=({{\rm{C}}}_{{\rm{pre}}}-{{\rm{C}}}_{\mathrm{post}-\mathrm{corr}})/\,\mathrm{Ln}({{\rm{C}}}_{{\rm{pre}}}-{{\rm{C}}}_{\mathrm{post}-\mathrm{corr}})$$
- *Total mass of albumin loss* was detected in dialysate (from PDC or TDC) according to the following equation:

$${\rm{Albumin}}\,{\rm{loss}}={{\rm{C}}}_{{\rm{albumin}}}\,\ast \,{{\rm{Q}}}_{{\rm{D}}}\,\ast \,{\rm{tHD}}$$

where Q_D_ represents dialysate flow and C_albumin_ the dialysate concentration of albumin. Total dialysate flow (Q_D_) includes ultrafiltration rate.

### Statistical analyses

Qualitative variables were expressed as number (percentages). Quantitative variables were expressed as mean (standard deviation, sd); median [min-max].

The differences between dialyzers and dialysis modalities were tested by two-way analysis of variance (ANOVA) after normality and equal variance were tested. When initial ANOVA indicated significant differences between the studied groups, post-hoc tests of multiple comparisons were performed for dialyzers effect whatever the mode was (p-values adjusted following the Bonferroni’s method).

Regarding the comparative quantification of dialysate albumin loss, a scatter of differences was visualized according to the Bland–Altman representation. Mean and limits of agreement, defined as mean ± 1.96 sd were computed.

Wilcoxon non parametric signed rank tests were used to test differences in dialysate albumin loss (with PDC only) within the subgroup of patients with use of the 4 dialyzers in post-dilution HDF.

Values were considered statistically significant at p < 0.05. All analyses were carried out with Statistical Package for Social Sciences version 18.0 (IBM Inc, USA).

## Supplementary information

Consort Checklist

Trial protocol

Trial complete protocol

Informed Consent Form
