# Supplementary material for: Randomised trial on clinical performances and biocompatibility of four high-flux hemodialyzers in two mode treatments: hemodialysis vs post dilution hemodiafiltration
Source: Sci Rep. 2019 Dec 4;9:18265. doi: 10.1038/s41598-019-54404-7 (PMC6892817; doi:10.1038/s41598-019-54404-7)
Supplement: Supplementary file 2 — Trial protocol [file 41598_2019_54404_MOESM2_ESM.pdf]

BIOMODAL trial protocol (NCT 03262272)

Title	Evaluation of Biocompatibility and Performances of 4 Dialyzers in Different Mode Treatments (BIOMODAL)<br>ClinicalTrials.gov Identifier: NCT03262272, First posted: 25/08/2017
Sponsor	Hemotech SAS, 19 avenue de l'Europe, CS 62270, 31522 Ramonville St Agne cedex, FRANCE
Coordinator of the project	Pr Jean-Paul CRISTOL, nephrologist, Department of Biochemistry and Hormonology, University Hospital Center of Montpellier, FRANCE<br>Tél: +33-467-33-83-15, email: jp-cristol@chu-montpellier.fr
Investigators	Caroline CREPUT, AURA, Paris, FRANCE.<br>Mouloud BOUZERNIDJ, Clinique Hemera, Yvetot, FRANCE.<br>Bruno SEIGNEURIC, University Hospital Center, Toulouse, FRANCE<br>Lotfi CHALABI, AIDER, Montpellier, FRANCE.
Aim of the study	This study aimed at evaluating the performances and biocompatibility of 4 different hemodialyzers in order to personalize device prescription according to dialysis treatment (HD or HDF) and patient profile.
Study design	Multicenter, prospective, randomized and comparative cross over study
Number of patients	32 patients
Duration of the study	Follow-up per patient: 4 weeks (one week per dialyzer)<br>Period of recrutment : 6 months<br>Inclusion start period: November 2016
Number of centers involved	4 (8 patients per center); 2 centers performing HD and the 2 others HDF treatment (see Fig. 1)
Inclusion criteria	❑ Adult patient with chronic kidney disease dialyzed for at least one month and treated with the modality used in the center (HD or HDF) for the study<br>❑ Regarding post-dilution HDF mode, patient treated with a minimum convective volume of 20L (cf EuDIAL guidelines)<br>❑ Patient treated with high permeability membrane, with high surface area dialyzer ≥ 1,8 m²<br>❑ Patient with vascular access allowing a blood flow rate with a minimum of 300 mL/min<br>❑ Patient covered by the social French health organism<br>❑ Patient informed of the study goals and having signed the informed consent
Exclusion criteria	❑ Patient with a vascular access not allowing a blood flow rate minimum of 300 mL/min<br>❑ Patient with a fast progressive chronic disease<br>❑ Patient with uncontrolled anemia<br>❑ Patient refusing to sign the informed consent<br>❑ Pregnant or nursing patient<br>❑ Pediatric patient<br>❑ Patient under tutorship

Primary objective	Evaluation of middle molecule (β2 microglobulin) extraction
Secondary objectives	- Evaluation of other middle or large molecule extraction:<br>myoglobin (17kDa), Beta Trace (21kDa), Free immunoglobulin light chains Kappa (23kDa), myostatin (40kDa), glycoprotein orosomucoïd (44kDa)<br>- Extraction of uremic toxins: urea, creatinin, inorganic phosphates (PO4)<br>- Evaluation of dialysis adequacy (Kt/V)<br>- Biocompatibility assessment: TNF-α and IL-6 (T0 et Tend)<br>- Monitoring of nutrition status:<br>1) quantification of albumin losses during the session in the used dialysate:<br>A comparative quantification of perdialytic albumin losses using either a " pull/push " syringe allowing to collect continuous spent sampling of dialysate (namely partial dialysate collection, "PDC") or the total dialysate collection (namely "TDC"), which represents the gold standard method to evaluate mass balances achieved during dialysis for a given solute, will be performed in a subgroup of patients (i.e. only 1 center), all being dialyzed with post-dilution HDF (n=8 patients for PDC and n=4 for TDC due to the cumbersome method).<br>2) dosage of serum albumin and transthyretin before the session<br>- Inflammation: CRP
Practical procedures	Eligible patients will be assigned to receive either post-dilution HDF (first two centers) or conventional high-flux HD (last two centers) for 4 weeks.<br>During this period, all patients will be sequentially dialyzed with 4 different dialyzers (one/week, the two first sessions of the week; the last session of the week being performed with the patient's own dialyzer and used as a wash out session): Leoceed 21HX, Polypure 22S+, Rexsys 27H and VIE-21A.<br>The sequence of dialyzers use will be randomly assigned: per center, device × week allocation scheme will be generated by random permutation of a block of 4 (computer generation). All patients in a given center will follow the scheme allocated to this center (see Fig. 1). The randomization sequence will be centralized and computed by the statistician<br>No run-in period before entering in the active study phase will be performed<br><br>Blood sample collection:<br>Blood samples will be drawn weekly during the midweek dialysis session from the arterial line before and after dialysis.<br>- Routine laboratory analyses including serum urea, creatinine and PO4 (all being evaluated pre- and post-dialysis) will be locally performed.<br>- Specific biomarkers including serum β2 microglobulin, myoglobin, Beta Trace, Free immunoglobulin light chains Kappa, myostatin, glycoprotein orosomucoïd, CRP, TNF-α, IL-6, albumin, transthyretin will be performed on samples collected weekly, centrifuged, aliquoted, frozen and further analyzed in a central laboratory.

Centers	Dialysis modality	Week 1	Week 2	Week 3	Week 4
Center 1 (n=8)	post dilution HDF	Rexsys 27 H	VIE-21A	Polypure 22 S+	Leoceed 21 HX
Center 2 (n=8)	post dilution HDF	Leoceed 21 HX	Rexsys 27 H	VIE-21A	Polypure 22 S+
Center 3 (n=8)	HD	Polypure 22 S+	VIE-21A	Leoceed 21 HX	Rexsys 27 H
Center 4 (n=8)	HD	Leoceed 21 HX	VIE-21A	Rexsys 27 H	Polypure 22 S+

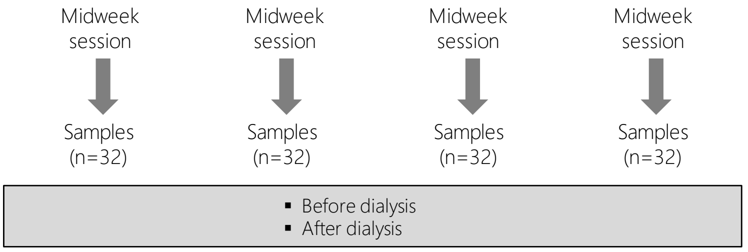

Fig. 1. Study design and sequences of randomization per center

Statistical analysis

The differences between dialyzers and dialysis modalities will be tested by two-way analysis of variance (ANOVA) after normality and equal variance will be tested. When initial ANOVA will indicate significant differences between the studied groups, post-hoc tests of multiple comparisons will be performed for dialyzers effect whatever the mode will be (p-values adjusted following the Bonferroni's method).

Regarding the comparative quantification of dialysate albumin loss, a scatter of differences will be visualized according to the Bland–Altman representation. Mean and limits of agreement, defined as mean±1.96 sd will be computed.

Wilcoxon non parametric signed rank tests will be used to test differences in dialysate albumin loss (with PDC only) within the subgroup of patients with use of the 4 dialyzers in post-dilution HDF.

Values will be considered statistically significant at $p<0.05$. All analyses will be carried out with Statistical Package for Social Sciences version 18.0 (IBM Inc, USA).
