# Supplementary material for: Randomised trial on clinical performances and biocompatibility of four high-flux hemodialyzers in two mode treatments: hemodialysis vs post dilution hemodiafiltration
Source: Sci Rep. 2019 Dec 4;9:18265. doi: 10.1038/s41598-019-54404-7 (PMC6892817; doi:10.1038/s41598-019-54404-7)
Supplement: Supplementary file 3 — Trial complete protocol [file 41598_2019_54404_MOESM3_ESM.pdf]

BIOMODAL

EVALUATION OF THE BIOCOMPATIBILITY AND PERFORMANCE OF HEMODIALYZERS USED IN DIFFERENTES DIALYSIS MODALITIES: CONVENTIONAL HEMODIALYSIS AND POST-DILUTION HAEMODIAFILTRATION

Biomedical Research
N° 2016-A01122-49

Coordinator

Professor Jean-Paul CRISTOL
Nephrologist
CHU Lapeyronie
Pôle Biologie Pathologie – Département de Biochimie
371 ave du Doyen Giraud
34295 MONTPELLIER cedex, FRANCE

Sponsor

HEMOTECH SAS
19 Avenue de l'Europe
CS 62270
31522 RAMONVILLE SAINT AGNE Cedex, FRANCE

Summary

1 PROJECT SUMMARY 4
2 GENERAL INFORMATION 4
3 RATIONALE AND BACKGROUND 5
4 STUDY GOALS AND OBJECTIVES 8
4.1 Primary objective 9
4.2 Secondary objectives 9
5 PATIENTS 9
5.1 Inclusion criteria 9
5.2 Non inclusion criteria 9
5.3 Exit criteria 10
5.4 Description of the definitive or temporary shutdown rules 10
5.5 Management arrangements after the study 10
6 DEVICES 10
7 METHODOLOGY 11
7.1 Study design, dialyzers and dialysis conditions 11
7.2 Duration of the study 12
7.3 Information and consent procedures 13
7.4 Samples 13
7.5 Follow-up of the study 14
7.5.1 Eligibility criteria 14
7.5.2 History of the patient 14
7.5.3 Case Report Form – Clinical criteria 15
7.5.4 Data collection 15
7.5.5 Anonymity 15
7.6 Associated Drugs 15
8 DATA TO COLLECT AND ANALYSIS TO PERFORM 15
9 STATISTICAL ANALYSIS 17
9.1 Calculation of the sample size 17

9.2 Principle of the analyzes 17
10 ADVERSE EVENTS AND NEW EVENTS 18
10.1 Adverse events 18
10.2 Serious Adverse Events 18
10.3 Collection of information and procedure for reporting a serious AE 18
10.4 New events 19
11 RIGHT OF ACCESS TO DATA AND ORIGINAL SOURCE DOCUMENTS 19
12 QUALITY CONTROL AND QUALITY ASSURANCE 19
13 ESTABLISHING AN INDEPENDENT MONITORING COMMITTEE 19
14 ETHICAL EVALUATION OF THE SPECIAL MONITORING PROCEDURES PROVIDED BY THE PROTOCOL 19
15 DATA PROCESSING AND STORAGE OF DOCUMENTS AND DATA RELATED TO THE RESEARCH 19
16 RULES RELATING TO PUBLICATION 20
17 REFERENCES 20
18 APPENDICES 23

1 PROJECT SUMMARY

Objectives of this prospective multicenter randomized cross-over study are to evaluate the influence of two dialysis treatments (HD vs post-dilution HDF) with four high-flux (polysulfone or polyethersulfone) dialyzers i) in the clearance evaluation for small, middle and large molecular weight substances and ii) in the modification of several biological responses including parameters of inflammation and nutrition. In addition, a comparative quantification of perdialytic albumin losses using either a partial collection of continuous spent dialysate or the total dialysate collection will be performed in a subgroup of patients.
Primary outcome will be β2-microglobulin removal. Secondary outcomes will be i) extraction of other uremic solutes ii) parameters of inflammation and nutrition and iii) comparative quantification of perdialytic albumin losses.
32 chronic kidney disease patients treated with extrarenal replacement therapy and issued from 4 dialysis centers will be assigned to receive either post-dilution HDF (2 first centers) or conventional high-flux HD (2 other centers) for 4 weeks. During this period, all patients will be sequentially dialyzed with 4 different dialyzers (one/week): Leoceed 21HX, Polypure 22S+, Rexsys 27H and VIE-21A. The sequence of dialyzers use will be randomly assigned. All patients in a given center will follow the scheme allocated to this center.
Dialysis conditions will remain unchanged for each patient: 3 sessions/week, 3-4 hours/session, blood flow (QB) of 300-400mL/min, ultrapure bicarbonate buffered dialysate, dialysate flow (QD) in the range 500-700mL/min.
Blood samples will be drawn weekly during the midweek dialysis session before and after dialysis. Routine laboratory analyses including serum urea, creatinine and PO4 (all being evaluated pre- and post-dialysis) will be locally performed. Specific biomarkers including serum β2-microglobulin, myoglobin, free light chain κ, Beta Trace Protein, myostatin, orosomucoid, CRP, TNF-α, IL-6, albumin and transthyretin will be performed on frozen samples in a central laboratory.
Results of this study should bring additional information on the performance and biocompatibility of the 4 hemodialyzers tested and help in personalizing device prescription according to dialysis treatment (HD or HDF) and patient profile.

2 GENERAL INFORMATION

Title

Evaluation of the biocompatibility and performance of hemodialyzers used in different dialysis modalities: conventional hemodialysis and post-dilution haemodiafiltration

ClinicalTrials.gov Identifier

NCT03262272, First posted: 25/08/2017

Sponsor

HEMOTECH SAS
19 Avenue de l'Europe – CS 62270

31522 RAMONVILLE SAINT AGNE Cedex

Phone : +33 5 61 75 27 27
Fax : +33 5 61 75 00 43
E-mail : info@hemotech.fr

Coordinator of the project

Pr Jean-Paul CRISTOL, nephrologist
Department of Biochemistry and Hormonology
University Hospital Center of Montpellier
FRANCE

Phone: +33 4 67 33 83 15
Fax : +33 4 67 33 83 93
E-mail: jp-cristol@chu-montpellier.fr

Investigators

Caroline CREPUT, AURA, Paris, FRANCE.
Mouloud BOUZERNIDJ, Clinique Hemera, Yvetot, FRANCE.
Bruno SEIGNEURIC, University Hospital Center, Toulouse, FRANCE
Lotfi CHALABI, AIDER, Montpellier, FRANCE.

Clinical laboratory

Department of Biochemistry and Hormonology
University Hospital Center of Montpellier
FRANCE

3 RATIONALE AND BACKGROUND

With the aging population, the number of patients receiving chronic dialysis treatment keeps increasing with an average age around 75 years old.

Role of dialysis
Dialysis allows the elimination of toxins of variable molecular weight.
Molecules to be purified can be schematically divided according to their molecular weight into
- Water-soluble small molecules with a molecular weight below 500 Da
These small water-soluble molecules, including urea, are easily removed by all dialysis techniques by diffusion phenomenon.
- Middle molecules of molecular weight greater than 500 Da
These middle molecules have deleterious effects and must be removed, even though a complete removal is difficult to reach. Along with diffusion phenomenon, convection method has to be performed. First middle molecule described was ß2microglobulin, which is known to cause amyloidosis, an inflammatory disease with high concentrations of plasma ß2microglobulin, in dialysis patients.

European Uremic ToXin Work Group (EUToX) has studied more than 850 publications and found 90 molecules with proven accumulation and deleterious effects in chronic kidney disease patients. The range of concentration is very wide, ranging from ng/L to g/L, and with degree of toxicity not necessarily related to its concentration. [4]

Consequences of dialysis: the inflammatory syndrome

The chronic microinflammatory state observed in advanced CKD (including dialysis) patients has been widely described and of multifactorial origin.

Some factors are directly related to the patient: kidney disease, associated co-morbidities, oxidative stress, infections, obesity and genetic or immunological factors. Others causing the chronic inflammatory syndrome are the direct consequences of the extracorporeal circuit including dialysis quality and biocompatibility of the membranes [1,2].

In CKD patients, inflammation is partly related to the degradation of cellular proteins, secondary to the toxicity of hyper-uremia [3]. Other factors may also be involved, such as decreased renal clearance of cytokines or sodium and fluid overload [2].

Dialysis techniques: conventional hemodialysis or haemodiafiltration

Currently two dialysis techniques are mainly used in France: conventional hemodialysis (HD) favoring the physico-chemical principle of diffusion and hemodiafiltration (HDF) combining both diffusion and convection.

The superiority of post-dilution HDF compared to HD for the removal of middle molecules is largely documented and explained by the contribution of convective transport to the global transfer of solutes, all the more important when molecular weight is high. Increase in convective transports in HDF has been associated with decreased inflammation and oxidative stress, improving nutritional status, anemia and quality of life of patients [5,6,7,8,9].

A recent Spanish study showed a 30% reduction in all-cause mortality when patients were treated with post-dilution HDF using convective volumes greater than 23L compared to conventional HD [6].

In order to optimize the post-dilution HDF and to obtain clinical benefits, the EUDIAL group recommended convective volumes around 20-22L per session [10].

These results on mortality are not confirmed by other studies [11,12,13]. Several preliminary studies have failed to shown any impact of HDF on nutritional criteria, or rather the decline of albumin (PM=66 kDa) and transthyretin (MW=55 kDa) levels with the technique. Such decreases can be attributable to variable amounts of per-dialytic protein leak depending on the membranes used, more or less permeable and therefore more or less effective in the elimination of other middle molecules (uremic or non-uremic toxins) [9].

Albumin losses can be measured in the dialysate by different sampling techniques such as "pull-push" syringe allowing to collect continuous spent sampling of dialysate or the total dialysate collection which represents the gold standard method to evaluate mass balances achieved during dialysis for a given solute [14].

Molecules to be removed

In order to evaluate performance of membranes, it is therefore interesting to verify the removal of middle molecules of increasing molecular weight.
• beta-2 microglobulin (11.8 kDa)
Pre-dialytic mean levels of serum beta-2 microglobulin were correlated with mortality in another analysis of the HEMO study [15].
• myoglobin (17 kDa), *a marker of middle molecules in haemodiafiltration*
• beta trace protein (21 kDa), an independent risk predictor of cardiovascular mortality in hemodialysis patients [19]
• κ free light chain (23 kDa), *to be eliminated in multiple myeloma*
• myostatin (40 kDa), *also playing a role in muscle mass*
• Orosomucoid glycoprotein (44 kDa), *inflammatory protein*

In the inflammatory process observed in CKD, cytokines, other middle molecular weight proteins, also play a fundamental role [16]. In CKD patients and hemodialysis, a huge immune change promotes the establishment and maintenance of a chronic inflammatory state.
In dialysis, this inflammation apperas less marked in HDF than in conventional HD. [17]

<u>Cytokines in chronic inflammation of CKD patient</u>
In dialysis patient, the use of so-called biocompatible membranes has allowed reduction in chronic inflammation by decreasing the inflammatory response and by removing inflammatory molecules, including cytokines, during the sessions.
The study of cytokine clearance during dialysis session has been studied with different dialyzers in HDF and HD. Variations in the results could be partly explained by the short half-life of cytokines and their secretion before, during and after the session.

Two of these cytokines play a fundamental role in the inflammatory process in CKD patients [13]: IL-6 and TNF-α.

IL-6
Interleukin-6 is a 22 to 26 kDa protein that plays a predominantly pro-inflammatory role in the immune response. It is secreted by most immune cells, especially adipocytes, lymphocytes, fibroblasts and monocytes, in response to a physiopathological stimulus (production of endotoxins, TNF-α, IL-1 or oxidative stress).
Levels of IL-6 increase in patients with CKD, especially after dialysis sessions (hemodialysis or peritoneal dialysis). This can be explained by several factors:
- Bio-incompatibility of dialyzers favoring the production of cytokines
- Decreased renal clearance
- Increase of monocyte production
- Water and fluid overload
- Congestive heart failure

IL-6 appears to play a role in the inflammatory mechanism of atheromatous plaque formation. It is also involved in protein catabolism, participating in the occurrence of a wasting syndrome, and the appearance of undernutrition and sarcopenia.

TNF-α
As a pro-inflammatory cytokine of 17 kDa, TNF-α plays a key role in the regulation of pro and anti-inflammatory mediators.

The increase in urea in CKD patients is the main factor associated with a significant increase in TNF-α production. Obesity, congestive heart failure and insulin resistance are all associated factors.

<u>Other markers of inflammation of CKD</u>
C Reactive Protein
CRP is a protein that is increased during the inflammatory response, especially of infectious cause. Its plasma levels are correlated with increases in IL-6.

Albumin
Albumin, the main plasma protein, is a key nutritional parameter. The correction of hypoalbuminemia is essential during a chronic inflammatory syndrome.

Blood count and anaphylatoxin counts
Increase in leucocytes is mainly associated with an infectious, viral or bacterial state, and secondarily with some noninfectious inflammatory reactions.
During bioincompatibility reactions, the levels of anaphylatoxins are increased, attesting of the complement system activation.

Improved hemocompatibility of dialysis membranes has significantly reduced inflammatory biological responses from the blood-membrane interaction. Recent research highlights not only the materials used in the membrane but also its surface properties such as grafting of vitamin E on some polysulfone membranes [18].
Each membrane has its own performance and hemocompatibility.
Which high permeability membrane should be chosen for which patient?

The removal performance of these membranes should be determined under actual conditions of use in order to make the most judicious choice for patients.
We propose by this work to study 4 hemodialyzers that already have CE certification for this indication.
This study aims to better advise nephrologists in their choice of prescription.

4 STUDY GOALS AND OBJECTIVES

This study aims at evaluating the biocompatibility and performance of high permeability polysulfone or polyethersulfone membranes of different surfaces (2.1m², 2.2m² and 2.7m²) as well as a vitamine E-grafted polysulfone membrane.
The extraction performances will be evaluated according to the type of techniques: hemodialysis or post-dilution haemodiafiltration.
The evaluation of biocompatibility will allow to assess the potential anti-inflammatory action of each membrane.
This study will be conducted in accordance in accordance with the ethical standards of the institutional and/or national research committee and with the 1964 Helsinki declaration and its later amendments or comparable ethical standards.

4.1 Primary objective

- Evaluation of middle molecule (β2 microglobulin, 11.8 kDa) extraction

⇨ Primary outcome measure

β2 microglobulin reduction ratio

4.2 Secondary objectives

- Evaluation of other middle or large molecule extraction:
myoglobin (17kDa), Beta Trace (21kDa), Free immunoglobulin light chains Kappa (23kDa), myostatin (40kDa), glycoprotein orosomucoïd (44kDa)
- Extraction of uremic toxins: urea, creatinin, inorganic phosphates (PO4)
- Evaluation of dialysis adequacy (Kt/V)
- Biocompatibility assessment: TNF-α and IL-6 (T0 et Tend)
- Monitoring of nutrition status:
1) quantification of albumin losses during the session in the used dialysate:
A comparative quantification of perdialytic albumin losses using either a " pull/push " syringe allowing to collect continuous spent sampling of dialysate (namely partial dialysate collection, "PDC") or the total dialysate collection (namely "TDC"), which represents the gold standard method to evaluate mass balances achieved during dialysis for a given solute, will be performed in a subgroup of patients (i.e. only 1 center), all being dialyzed with post-dilution HDF (n=8 patients for PDC and n=4 for TDC due to the cumbersome method).
2) dosage of serum albumin and transthyretin before the session
- Inflammation: CRP

5 PATIENTS

5.1 Inclusion criteria

- ❑ Adult patient with chronic kidney disease dialyzed for at least one month and treated with the modality used in the center (HD or HDF) for the study
- ❑ Regarding post-dilution HDF mode, patient treated with a minimum convective volume of 20L (cf EuDIAL guidelines)
- ❑ Patient treated with high permeability membrane, with high surface area dialyzer ≥ 1,8 m^2
- ❑ Patient with vascular access allowing a blood flow rate with a minimum of 300 mL/min
- ❑ Patient covered by the social French health organism
- ❑ Patient informed of the study goals and having signed the informed consent

5.2 Non inclusion criteria

- ❑ Patient with a vascular access not allowing a blood flow rate minimum of 300 mL/min
- ❑ Patient with a fast progressive chronic disease
- ❑ Patient with uncontrolled anemia
- ❑ Patient refusing to sign the informed consent
- ❑ Pregnant or nursing patient
- ❑ Pediatric patient
- ❑ Patient under tutorship

5.3 Exit criteria

- Express request of the patient
- Medical decision
- Move to another dialysis center not participating in the study
- Transplantation
- Medication without prior agreement of the physician
- Death

5.4 Description of the definitive or temporary shutdown rules

The Health Authorities or the Sponsor may make the decision of a definitive or temporary stoppage of the research.
The Sponsor reserves the right to discontinue the trial if it considers that its continuation is no longer justifiable for medical or methodological reasons.

5.5 Management arrangements after the study

Patients will be dialysing under the usual conditions, required by their condition at the end of the research or in case of premature discontinuation of treatment or exclusion of the research. They will no longer be subject to specific reviews relating to research.

6 DEVICES

All medical devices are CE marked Class IIb hemodialyzers used in their indications.
All the characteristics, contra-indications and instructions for use are indicated in the instructions for use and these products must be used while respecting them.

- VIE A

Dialyzer	VIE 21A
Membrane	VItabranE, Vitamin E-grafted polysulfon Asahi
Surface	2,1m²
Ultrafiltration coefficient	89 ml/hxmmHg
Dimension of fibers	Thickness = 45 µm ; inner diameter = 185 µm
Sterilisation mode	Gamma rays in a humid environment

The Vitamine E-grafted polysulfone membrane, VItabranE™ (VIE) received 6 authorizations for biomedical research:

- Record N° Afssaps : 2006-A00187-44, H-D-vie-mrs
  Coordinator : Pr Michel OLMER, ATUP Marseille, FRANCE
- Record N° Afssaps : 2006-A00436-45, H-D-ViE
  Coordinator : Pr Bernard CANAUD, CHU Montpellier, multicenter study
- Record N° Afssaps : 2008-A00460-55, VIE-AGE
  Coordinator : Pr Philippe RIEU, CHU REIMS, Association ARPDD REIMS
- Record N° Afssaps : 2010-A00638-31, VIE-AP HP-2010
  Coordinator : Dr Séverine BEAUDREUIL, Bicêtre Hospital
- Record N° ANSM : 2012-A01502-41 ; EVIA 2012
  Coordinator : Pr JP Cristol, CHU Montpellier

- Record N° ANSM : 2015-A01391-48 ; EVA.D 2015
  Coordinator : Dr Frangié, Bordeaux Nord Clinic

And one authorization for routine care research:

- Record N° Afssaps : 2011-A00303-38, VIE AURAD
  Coordinator : Docteur Philippe CHAUVEAU, Association AURAD Aquitaine

- LEOCEED 21HX

Dialyzer	Leoceed 21HX
Membrane	Polysulfone Asahi™
Surface	2,1 m^2
Ultrafiltration coefficient	94 ml/hxmmHg
Dimension of fibers	Thickness = 35 µm ; inner diameter = 200 µm
Sterilisation mode	Gamma rays in a dry environment

- REXSYS 27H

Dialyzer	Rexsys 27H
Membrane	Polyethersulfone
Surface	2,7 m^2
Ultrafiltration coefficient	124 ml/hxmmHg
Dimension of fibers	Thickness = 30 µm ; inner diameter = 200 µm
Sterilisation mode	Gamma rays in a dry environment

- POLYPURE 22S+

Dialyzer	Polypure 22S+
Membrane	Polysulfone
Surface	2,2 m^2
Ultrafiltration coefficient	74 ml/hxmmHg
Dimension of fibers	Thickness = 40 µm ; inner diameter = 200 µm
Sterilisation mode	Steam

7 METHODOLOGY

7.1 Study design, dialyzers and dialysis conditions

This study is a prospective multicenter randomized comparative cross-over trial.
Four different dialysis facilities (AURA Paris, Yvetot clinic, AIDER clinic and Toulouse university hospital center) will be involved in the study.
Eligible patients will be assigned to receive either post-dilution HDF (first two centers) or conventional high-flux HD (last two centers) for 4 weeks. During this period, all patients will be sequentially dialyzed with 4 different dialyzers (one/week, the two first sessions of the week; the

last session of the week being performed with the patient's own dialyzer and used as a wash out session): Leoceed 21HX, Polypure 22S+, Rexsys 27H and VIE-21A.
The sequence of dialyzers use will be randomly assigned: per center, device × week allocation scheme wil be generated by random permutation of a block of 4 (computer generation). All patients in a given center will follow the scheme allocated to this center. The randomization sequence will be centralized and computed by the statistician.
No run-in period before entering in the active study phase will be performed

Dialysis sessions will be conducted according to usual procedures of the center, with increased monitoring and control of the patients treated with the tested hemodialyzers.
Patients will be treated in HD or post-dilution HDF, according to the usual conditions, specific to each patient. The frequency of dialysis (3 sessions per week), the duration of dialysis (3- 4h), the blood flow of the extracorporeal blood circuit (≥ 300 mL/min), the type (dialysate bicarbonate or other) and the dialysate flow (700 mL/min) will remain unchanged or will be documented in case of modification.
In case of need for adjustment, or changes in prescription of the medication, these changes will be documented. The modifications will respect recommendations.

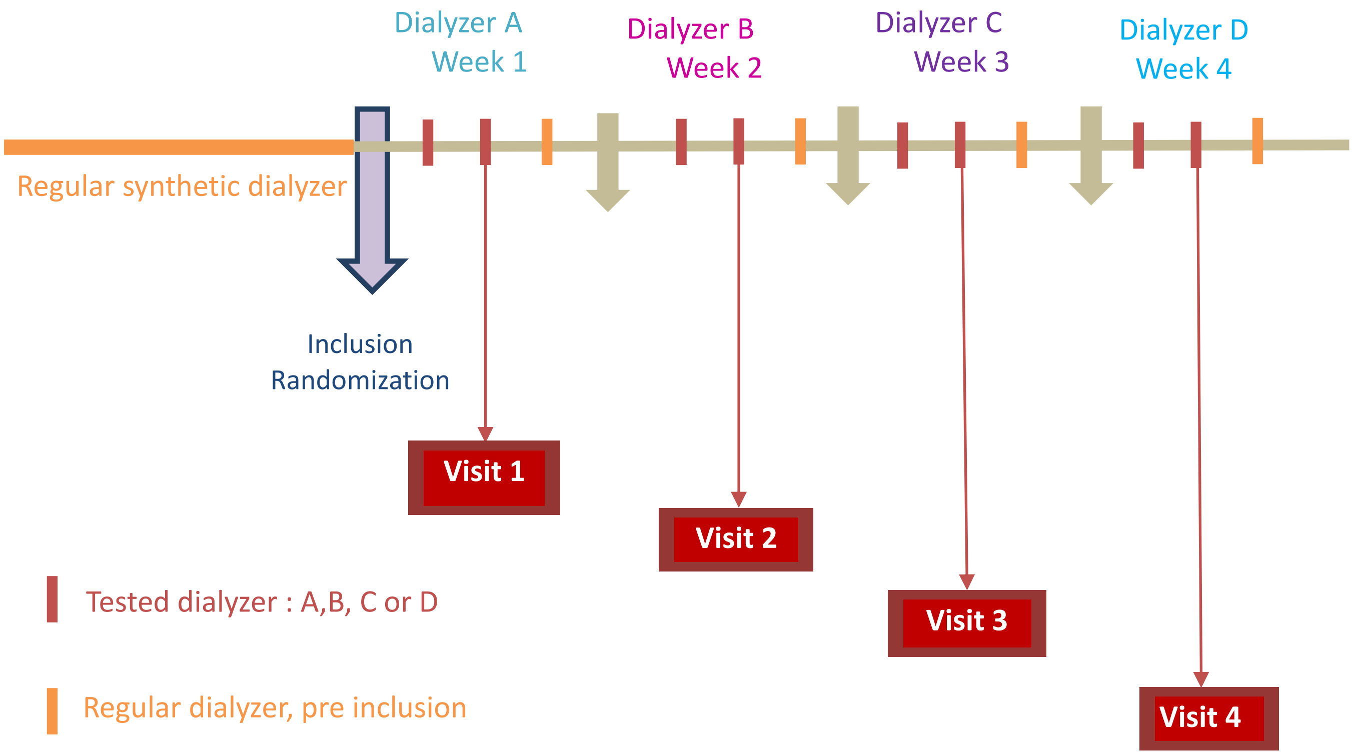

7.2 Duration of the study

Duration of the study: 4 weeks.
Period of recruitment: 6 months.
Inclusion start period: November 2016.

7.3 Information and consent procedures

Participation in the study will be offered to all 5D CKD patients followed for their extra-renal replacement therapy in one of the 4 investigation centers.

Within each center, after analyzing the medical records of all the patients, each investigator will propose to the eligible patients (compliance with the inclusion and non-inclusion criteria) to participate in the clinical trial by informing them of the completeness of the clinical trial.

An information letter will be given to the patient and commented with the investigator nephrologist. In the event of a favorable response, a visit will be scheduled to sign the consent (cf. Appendix 1) and start the study.

7.4 Samples

Blood samples needed for the study will be drawn weekly (for a total of 4 weeks) during the midweek session from the arterial line of the extracorporeal circuit without additional puncture before and after dialysis.

Each withdrawal will include:

<u>Tubes needed for local analyses</u>

Before dialysis

Type of tube	Volume	Parameters
1 EDTA tube	2,5 mL	Blood count
1 heparinized tube	2,5 mL	Urea, Creatinine, Phosphates

After dialysis

Type of tube	Volume	Parameters
1 EDTA tube	2,5 mL	Blood count
1 heparinized tube	2,5 mL	Urea, Creatinine, Phosphates

<u>Tubes needed for centralized analyses at the CHU of Montpellier</u>

The following tubes will be centrifuged, and serum and plasma aliquoted, frozen and further analyzed in the central laboratory.

Before dialysis

Type de tube	Volume	Parameters	Nb of aliquots	Volume of serum/plasma
1 heparinized tube	5 ml	CRP	1	500 µl
		Myoglobin		
		Beta trace	1	500 µl
1 dry tube	7 ml	Orosomucoid	1	500 µl
		Albumin		
		Transthyretin		
		Beta2 microglobulin		
		Free light chains kappa	1	500 µl
		C3a	1	150 µl
		C5a		
		TNF-α	1	500 µl
		IL-6		
		Myostatine	1	150 µl
TOTAL before dialysis	12 ml			

After dialysis

Type de tube	Volume	Parameters	Nb of aliquots	Volume of serum/plasma
1 heparinized tube	5 ml	CRP	1	500 µl
		Myoglobin		
		Beta trace	1	500 µl
1 dry tube	7 ml	Orosomucoid	1	500 µl
		Beta2 microglobulin		
		Free light chains kappa	1	500 µl
		C3a	1	150 µl
		C5a		
		TNF-α	1	500 µl
		IL-6		
		Myostatin	1	150 µl
TOTAL after dialysis	12 ml			

7.5 Follow-up of the study

A Case Report Form (CRF) will be completed for each patient included in the study. This document contains different sheets: Eligibility Criteria, Patient History, and, for each visit, a Data Collection sheet.

7.5.1 Eligibility criteria

The inclusion and exclusion criteria are to be fulfilled by the Investigator.

7.5.2 History of the patient

Items to collect are as follows:

• Patient data: gender / age / date of start dialysis / dry weight

• Characteristics of dialysis session: number of sessions per week / name of the dialyzer used before inclusion in the study

• Technique used: Post-dilution HDF or HD with session parameters
• Characteristics of vascular access: type (arteriovenous fistula/loop/catheter); date of implantation-creation
• Medical and surgical history, cardiovascular risk factors: diabetes (and type); hypertension; dyslipidemia; smoking habits; ischemic heart disease
• Therapeutic class of erythropoietin stimulating agents, type of administration (intravenously or subcutaneously) and initiation date.
• Iron administration and type of administration (orally, intravenously or subcutaneously) and initiation date.

7.5.3 Case Report Form – Clinical criteria

Follow-up of the patient during the study

During the follow-up, the following data will be collected:
• Dry weight / weight before and after dialysis / blood flow / dialysate flow / convective volume
• Possible therapeutic modifications
• Intercurrent pathology (s)
• Information on priming and restitution

7.5.4 Data collection

This sheet contains the various parameters to evaluate and to record and must be filled on the day of the assessments.

7.5.5 Anonymity

The subjects participating in the study will be coded so that their anonymity is guaranteed. They will be identified by the first three letters of their name, the first letter of their first name and the number of the center.
The Case Report Forms are identified as follows:
CRF XXX - Y BIOMODAL zzz

XXX is the first 3 letters of the patient's name.
Y is the first letter of the patient's first name.
ZZZ are the first 3 letters of the name of the center

The Investigator will assign himself the identifier (XXX-Y) of the CRF of each of his patients, identifying that he will have to fill in the sheet "Inclusion". There will be one file per patient; the identification of the CRF will remain the same for the duration of the study.

7.6 Associated Drugs

Any therapeutic modification will be informed.

8 DATA TO COLLECT AND ANALYSIS TO PERFORM

	Dialyzer A week 1	Dialyzer B week 2	Dialyzer C week 3	Dialyzer D week 4
Parameters of the session				
Dry weight	x	x	x	x
Weight before dialysis	x	x	x	x
Weight after dialysis	x	x	x	x
Duration of the session	x	x	x	x
Blood flow	x	x	x	x
Dialysate flow	x	x	x	x
Convective volume	x	x	x	x
KT/V	x	x	x	x
Quality of priming	x	x	x	x
Quality of restitution	x	x	x	x
Routine biological parameters				
Hemoglobin pre-dialysis	x	x	x	x
Hematocrit pre-dialysis	x	x	x	x
Platelets pre-dialysis	x	x	x	x
Leukocytes pre-dialysis	x	x	x	x
Urea pre- and post-dialysis	x	x	x	x
Creatinine pre- and post-dialysis	x	x	x	x
Phosphates pre- and post-dialysis	x	x	x	x
Specific biological parameters				
Inflammation & nutrition				
Albumin pre-dialysis	x	x	x	x
Transthyretin pre-dialysis	x	x	x	x
CRP pre-dialysis	x	x	x	x
Middle molecules				
β2 microglobulin pre- and post-dialysis	x	x	x	x
Myoglobin pre- and post-dialysis	x	x	x	x
Beta trace pre- and post-dialysis	x	x	x	x
Free light chains kappa pre- and post-dialysis	x	x	x	x
Myostatin pre- and post-dialysis	x	x	x	x
Orosomucoid pre- and post-dialysis	x	x	x	x
Markers of biocompatibility				
TNF-α pre- and post-dialysis	x	x	x	x
IL-6 pre- and post-dialysis	x	x	x	x

The hematological parameters being part of the monthly routine biology of dialysis patients, the dosages will be carried out locally by the laboratory of the dialysis center.

All other tubes will be centrifuged, aliquoted and frozen at -20 °C for further determination in a central laboratory (Montpellier). Albumin, transthyretin and CRP assays will also be performed in Montpellier to avoid any bias related to the assay techniques.

9 STATISTICAL ANALYSIS

9.1 Calculation of the sample size

This study has mainly a pragmatic exploratory orientation. It aims to try to detect differences in performance between dialyzers tested whose importance is clinically relevant. To do this, it was considered that, for the measured variables, a Cohen effect size of about 0.6 in two-to-two comparisons would meet this criterion of relevance. Under these conditions, in a bilateral situation and setting a 5% alpha risk and a 20% beta risk, a minimum of 32 patients is required.

General remark

The design of this study involves the comparison of 4 dialyzers for a same patient over 4 weeks. Each week begins with the use of one studied dialyzer over 2 sessions, the second session serving as a reference. Then, the third session of the week is performed with the usual dialyzer. On this basis, the participating nephrologists have considered that any risk of persistent effect (carry-over) could be excluded and that the duration of the study was short enough to rule out a period effect related to a change in the subject's clinical status (no effect period). In addition, a randomization of the Latin square type was not considered possible for practical organization reasons at each center level.

As a result, a random order of dialyzer use was decided once (random switching of 4 elements) and this order will be applied for all patients of a same center: all patients in a given center will follow the scheme allocated to this center.

The sequence of dialyzers use will be randomly assigned: per center, device × week allocation scheme will be generated by random permutation of a block of 4 (computer generation).

9.2 Principle of the analyzes

The included population will be described in terms of demographic and medical inclusion data.

Two populations of analysis are planned: the global population including all the patients with at least one dialyzer tested and the per-protocol population (PP) including all the subjects having tested the 4 dialyzers.

The main analyzes will be conducted on the PP population and additional sensitivity analyzes on the overall population.

The main analyzes will use a univariate generalized linear model integrating the variable tested as a dependent variable and the standard hemodialysis factor (2 levels: conventional HD or HDF), the dialyzer factor (4 levels) and the center factor (4 levels) as fixed factors. A full factorial model will be applied. Two-to-two comparisons will then be conducted by adjusting the alpha risk according to the Bonferonni method.

All analyses will be carried out with Statistical Package for Social Sciences version 18.0 (IBM Inc, USA).

10 ADVERSE EVENTS AND NEW EVENTS

10.1 Adverse events

An adverse event (AE) is an event occurring in a subject during the clinical trial. It may be a change in the condition of the subject or biological findings that has or could have an adverse effect on the health or well-being of the subject.
All AE will have to be recorded in the CRF. The absence of AE during the relevant period should also be mentioned.
The investigator should describe clearly AE and specify the nature of AE as well as details about it.

10.2 Serious Adverse Events

An adverse event is considered serious because of the rating that the Investigator attributes to him on the scale of intensity. An adverse event is considered serious (SAE) because of its nature and whatever the intensity of the symptoms that compose it.
Serious adverse events by nature are:
- death,
- hospitalization or prolongation of hospitalization,
- life-threatening situation,
- cancer,
- overdose,
- permanent or temporary disability, total or partial,
- congenital anomaly.

10.3 Collection of information and procedure for reporting a serious AE

Serious adverse events should be reported via the adverse event reporting form.
These serious adverse events should be reported by telephone or fax within one working day by the Principal Investigator to the Sponsor of the study, irrespective of how long it may take to event occurred and the moment the investigator became aware of it for the first time.

Name / Address of the Sponsor<br>HEMOTECH SAS<br>19 avenue de l'Europe CS 62270<br>31522 Ramonville Saint Agne cedex	Phone : +33 5 61 75 27 27<br>Fax : +33 5 61 75 00 43

The Serious Adverse Event Reporting Form should be completed, signed and sent to the study monitor within five working days.
The Sponsor may request further information from the Investigator and re-form the Report Form for computerized exploitation by his specialized department, adding the statement of the Investigator.
The reporting of a serious adverse event to the ANSM is the responsability of the Promoter.
The Ethics comittee should be informed of the occurrence of any serious adverse event.

10.4 New events

If a new event is likely to affect the safety of the persons who are suitable for the research, the Sponsor and the Investigator shall take the appropriate urgent security measures. The Sponsor shall promptly inform the competent authority and the Ethics Comittee of such new events and, if appropriate, of the measures taken.

11 RIGHT OF ACCESS TO DATA AND ORIGINAL SOURCE DOCUMENTS

In compliance with Article L.1122-1 of the Public Health Code, the patient may be informed, during or after the research, of his health, information which is held by the Investigator or the physician who follows him. He will be informed, at his request, of the overall results of the trial by the investigator.

12 QUALITY CONTROL AND QUALITY ASSURANCE

In order to check the compliance of the study with what has been defined in the protocol, a monitoring of inclusions and data (patient data and biological data) will be implemented.
A process of data collection, entry, control, check, correction and analysis is initiated by the sponsor using an ARC agency. He (she) will allow the patient's information to be checked, to check the update of the patient follow-up (request for additional information by monitoring sheet, corrections, etc.) and to monitor more generally the progress of the protocol.
This quality control will be carried out by the Sponsor.
Throughout the study, the collected data will be checked and the investigator undertakes to send to the Sponsor the patient's CRF duly completed, and to respond to requests for supplement data or information check.

13 ESTABLISHING AN INDEPENDENT MONITORING COMMITTEE

It is not planned to set up an independent monitoring committee. Data are collected by a dedicated person, responsible for the smooth logistical progress of the study. Data will be analyzed by an independent external body specialized in medical statistics. These results will then be forwarded to the study coordinator for interpretation.

14 ETHICAL EVALUATION OF THE SPECIAL MONITORING PROCEDURES PROVIDED BY THE PROTOCOL

No special monitoring is planned.
Patients will be followed up according to the usual criteria for monitoring hemodialysis patients.

15 DATA PROCESSING AND STORAGE OF DOCUMENTS AND DATA RELATED TO THE RESEARCH

The anonymized clinical data will be collected and their relevance will be verified by the Sponsor.
A process of data collection, control, check, correction and analysis is set up as part of the study's monitoring.

The statistical processing will be done by an independent body outside the Sponsor.

The sponsor and the investigator of a biomedical research must keep the documents and data related to this research for at least fifteen years after the end of the research or its early termination.

16 RULES RELATING TO PUBLICATION

The documents submitted during the evaluation as well as the results of this study are the exclusive property of the Sponsor.

In accordance with Article R 5121-13 of the Public Health Code, all investigators and any person who will have to collaborate in the trial are subject to professional confidentiality, especially regarding the nature of the studied products, the tests, the patients recruited and the results obtained. They may not, without the consent of the sponsor, give information related to the trial except to the Minister of Health, to the physicians and pharmacists inspectors of public health, to the General Director and to the Inspectors of the ANSM.

Any publication by the coordinator or by the investigators concerning results of this evaluation can only be carried out with the authorization of the Sponsor.

17 REFERENCES

1- Jofré R. et al
Inflammatory Syndrome in Patients on Hemodialysis.
JASN, S274–S280, 2006

2- Kaysen G.A.
Biochemistry and Biomarkers of Inflamed Patients: Why Look, What to assess.
Clin J Am Soc Nephrol, S56–S63, 2009

3- Galli F.
Protein damage and inflammation in uraemia and dialysis patients.
Nephrol Dial Transplant, v20–v36, 2007

4- Vanholder R. et al
Review on uremic toxins: Classification, concentration, and interindividual variability
Kidney International, Vol. 63, pp. 1934–1943, 2003

5- Canaud B. et al
Mortality risk for patients receiving hemodiafiltration versus hemodialysis
European results from the DOPPS. Kidney Int, 69:2087-2093, 2006

6- Maduell F. et al
High-Efficiency Postdilution Online Hemodiafiltration Reduces All-Cause Mortality in Hemodialysis Patients
JASN, 24(3):487-497, March 2013

7- Carracedo J. et al
On-Line hemodiafiltration reduces the proinflammatory $CD14^{+}CD\ 16^{+}$ Monocyte-Derived Dendritic Cells: A prospective, Crossover Study

JASN, 17(8):2315-2321, August 2006

8- Savica V. et al
Nutritional status in hemodialysis patients: options for on-line convective treatment
J Ren Nutr, 16(3):237-40, July 2006

9- Kantartzi K. et al
Can dialysis modality influence quality of life in chronic hemodialysis patients? Low-flux hemodialysis versus high-flux hemodiafiltration : a cross-over study
Ren Fail, 35 (2):216-21, 2013

10- Mostovaya I.M. et al.
High convection volume in online post-dilution haemodiafiltration: relevance, safety and costs
CKJ. 8(4): 368-73, August 2015

11- Grooteman M.P.C. et al
Effect of Online Hemodiafiltration on all-cause mortality and cardiovascular outcomes JASN, 23(6):1087-1096, June 2012

12- Mazairac A.H. et al
Effect of hemodiafiltration on quality of life over time
CJASN, 07; 8(1):82-9, January 2013

13- Vilar E. et al
Long-term outcomes in online hemodiafiltration and high-flux hemodialysis: a comparative analysis
CJASN, 4(12):1944-53, December 2009

14- Argiles A. et al
Precise quantification of dialysis using continuous sampling of spent dialysate and total dialysate volume measurement
Kidney Int, 52(2): 530-7, August 1997

15- Cheung A.K. et al
Serum β-2 Microglobulin Levels Predict Mortality in Dialysis Patients: Results of the HEMO Study
JASN, 17(2): 546-55, February 2006

16- Stenvinkel P. et al
IL-10, IL-6, and TNF-α: Central factors in the altered cytokine network of uremia—The good, the bad, and the ugly
Kidney Int, 1216–1233, 2005

17- Leurs P. et al
Effects of hemodiafiltration on uremic inflammation
Blood Purif. 35 Suppl 1: 11-7, 2013

18- Kokubo K. et al.
Evaluation of the biocompatibility of dialysis membranes
Blood Purif, 40:293–297, 2015

18 APPENDICES

APPENDIX 1

<u>Informed consent form (english version)</u>

**Mr – Mrs (NAME – First Name)**	
**Date of Birth :**	
**Address :**	

Dr. ________________________ proposed to me to participate in a biomedical research aiming to evaluate the biocompatibility and performances of 4 hemodialysers based on polysufone membranes, one of which being grafted with Vitamin E, during a period of 4 weeks. The goal of this study is to find the most suitable dialyzer for the dialysis technique and for the patient's profile.
The title of this study BIOMODAL, referenced under the number n ° 2016-A01122-49 is:
Evaluation of the BIOcompatibility and performance of hemodialysers used in different dialysis modalities.

This biomedical research is carried out according to the articles of the Public Health Code, in application of the decree n ° 2006-4777 of April 26th, 2006; this article has modified chapter 1 of title II of book 1 of the 1st part of the Public Health Code relating to biomedical research.
In accordance with this law, the Ethics Committee Sud-Méditerranée 1 has studied this research project and has issued a favorable opinion for its realization on 22/09/2016 under the following registration number: CPP 16 77 / 2016-A01122-49.

The Promoter of this study is the company HEMOTECH SAS - 19 Avenue of Europe - CS 62270 - 31522 RAMONVILLE SAINT AGNE Cedex.
The Research Coordinator is Professor Jean-Paul Cristol - CHU Lapeyronie Montpellier- 371 ave du Doyen GIRAUD – 34295 MONTPELLIER cedex.

This research is the subject of insurance contract no. 7224151204 with AXA France.

I accept freely and voluntarily to participate in this research, according to the conditions defined in the newsletter; I certify to be affiliated or beneficiary of a French social security system.

I also understood that I could, at any time during the study, withdraw my consent and inform my doctor without having to justify it, without incurring any responsibility or prejudice of this fact, without being penalized (e) and continuing to receive the best care available.

However, if I wish to withdraw my consent and thus leave the study, the data that have been obtained since my entry into the study until my withdrawal can be used in the general analysis of the study following the article 1122-1, clarifying the possibility of using the data collected from a patient who withdraws consent before the end of the study and gives the sponsor the opportunity to use this data.

I understood that I had a right of opposition on the use of the products of my body which had been collected, but that I could not benefit from any financial profit concerning the products marketed later from them.

I understood that I could at any time exercise the right of access, rectification and opposition guaranteed to me by articles 38, 39 and 40 of the Law n ° 2004-801 of August 6th, 2004 relative the protection of individuals with regard to the processing of personal data, and the computerized processing of personal data concerning me.

The conditions of my participation, including the duration of the participation, as well as the benefits and potential risks of the study in question, were clearly explained to me by Dr. ________________. I am well aware of the purpose of the study, the conditions of its realization and the constraints that result.
The risks involved with my usual dialysis treatment are negligible; the only difference is the use of another CE marked hemodialyzer.
The possible drawback of this study lies in the removal of two additional tubes before and after dialysis.

This consent does not relieve the sponsor and the investigators of their responsibilities.
An information letter was given to me; I had the opportunity to read it, understand it and keep it.
I acknowledge that I have been able to ask all the wished questions and to have received the satisfactory answers on all the desired information; I acknowledge that I have the opportunity to have any additional information that I may wish at any time.
I acknowledge that I have had sufficient time for reflection between this information and this consent, and have had the opportunity, if I wished, to discuss it with my doctor or relatives.
I acknowledge in particular that the right to have me assisted by a trusted person of my choice has been communicated to me.
I acknowledge that I have been informed that the study may be interrupted at any time by decision of the Promoter or the authorities and that all measures will be taken in this case to ensure my safety and, where appropriate, the continuation of my treatment.
I acknowledge that I have been informed that my personal participation in the study could be suspended if I did not follow the protocol.
I have understood that any new fact likely to call into question my consent to my participation in the study would be communicated to me.
I undertake to observe the constraints explained and specified in the information document, both to minimize the risks and for the proper implementation of the protocol.
In particular, I agree not to take any medicine other than those fixed by the study protocol without the authorization of the doctor who conducts the study or my doctor.
I will inform the doctor of the study as soon as possible if an unauthorized medication is taken.

I formally declare that I am not in a period of exclusion from other biomedical research and do not participate in any ongoing study.
I understood that hiding the truth can have detrimental consequences for my health. I therefore certify that I have responded truthfully to all the questions put to me, particularly those relating to my state of health and lifestyle.

I agree to respect the confidentiality rules applicable to the study, as explained to me beforehand.

Two copies of this consent have been completed; one copy was given to me, the other being for the doctor.

Done at .... on ....

Patient Signature

(Precede with "Read, understood and approved ")

Identification and contact information of the co-signer investigator
Signature of the Investigator
