# Supplementary material for: Randomised trial on clinical performances and biocompatibility of four high-flux hemodialyzers in two mode treatments: hemodialysis vs post dilution hemodiafiltration
Source: Sci Rep. 2019 Dec 4;9:18265. doi: 10.1038/s41598-019-54404-7 (PMC6892817; doi:10.1038/s41598-019-54404-7)
Supplement: Supplementary file 4 — Informed Consent Form [file 41598_2019_54404_MOESM4_ESM.pdf]

Consentement éclairé du patient

**M. – Mme (NOM – Prénoms)**
**Né(e) le :**
**Demeurant à :**

Le Docteur _________________________ m'a proposé de participer à une recherche biomédicale cherchant à évaluer la biocompatibilité et les performances de 4 hémodialyseurs possédant des membranes de la famille des polysulfones dont une est greffée à la Vitamine E, pendant une durée de 4 semaines. Le but est de trouver le dialyseur le plus adapté à la technique de dialyse utilisée ainsi qu'au profil patient.
Le titre de cette étude BIOMODAL, référencée sous le numéro n°2016-A01122-49 est :
Evaluation de la BIOcompatibilité et des performances d'hémodialyseurs utilisés dans différentes MODALités de dialyse.

Cette recherche biomédicale est conduite selon les articles du Code de la Santé Publique, en application du décret n°2006-4777 du 26 avril 2006; cet article a modifié le chapitre 1er du titre II du livre 1er de la 1ère partie du Code de la Santé Publique relatif aux recherches biomédicales.
Conformément à cette loi, le Comité de Protection des Personnes Sud Méditerranée 1 a étudié ce projet de recherche, et a émis un avis favorable à sa réalisation en date du 22/09/2016 sous le numéro d'enregistrement suivant : CPP 16 77 / 2016-A01122-49.

Le Promoteur de cette étude est la Société HEMOTECH SAS – 19 Avenue de l'Europe – CS 62270 – 31522 RAMONVILLE SAINT AGNE Cedex.
Le coordonnateur de la Recherche est le Professeur Jean-Paul Cristol – CHU Lapeyronie Montpellier – 371 ave du Doyen GIRAUD – 34295 MONTPELLIER cedex.

Cette recherche fait l'objet du contrat d'assurance n°7224151204 auprès de la Société AXA France.

J'accepte librement et volontairement de participer à cette recherche, selon les conditions définies dans la lettre d'information ; je certifie être affilié(e) ou bénéficiaire d'un régime français de Sécurité Sociale.

J'ai également compris que je pouvais, à tout moment pendant l'étude, retirer mon consentement et en informer mon médecin sans avoir à m'en justifier, sans encourir aucune responsabilité ni aucun préjudice de ce fait, sans en être pénalisé(e) et en continuant à recevoir les meilleurs soins disponibles.

Toutefois, si je souhaite retirer mon consentement et donc quitter l'étude, les données qui auront été obtenues depuis mon entrée dans l'étude jusqu'à mon retrait pourront être utilisées dans l'analyse générale de l'étude suite à l'article 1122-1 modifié clarifiant la possibilité d'utiliser les données collectées chez un patient qui retirerait son consentement avant la fin de l'étude et donne la possibilité au promoteur d'utiliser ces données.

J'ai bien compris que je disposais d'un droit d'opposition sur l'utilisation des produits de mon corps ayant fait l'objet d'une collecte, mais que je ne pouvais bénéficier d'aucun bénéfice financier concernant les produits commercialisés ultérieurement à partir d'eux.

J'ai bien compris que je pouvais à tout moment exercer le droit d'accès, de rectification et d'opposition qui m'est garanti par les articles 38, 39 et 40 de la Loi n°2004-801 du 6 août 2004 relative à la protection des personnes physiques à l'égard du traitement de données à caractère personnel, et relativement au traitement informatisé des données nominatives me concernant.

Les conditions de ma participation, notamment la durée de celle-ci, ainsi que les bénéfices et les risques éventuels de l'étude en question, m'ont été expliqués clairement par le Docteur ___________________. J'ai bien pris connaissance de l'objectif de l'étude, des conditions de sa réalisation et des contraintes qui en découlent.

Les risques encourus par rapport à mon traitement de dialyse habituel apparaissent négligeables ; la seule différence réside dans l'utilisation d'un autre hémodialyseur marqué CE.

L'éventuel inconvénient de cette étude réside dans le prélèvement de deux tubes supplémentaires avant et après dialyse.

Le présent consentement ne dégage pas le promoteur et les investigateurs de leurs responsabilités.

Une lettre d'information (Lettre d'information Patient) m'a été remise ; j'ai eu la possibilité de la lire, la comprendre et la conserver.

Je reconnais avoir pu poser toutes les questions souhaitées et avoir reçu les réponses satisfaisantes sur toutes les informations désirées ; je reconnais avoir la possibilité qui m'est offerte de disposer à tout moment des informations complémentaires que je pourrais souhaiter.

Je reconnais avoir disposé d'un temps de réflexion suffisant entre ces informations et le présent consentement, et avoir eu si je le souhaitais l'opportunité d'en discuter avec mon médecin ou mes proches.

Je reconnais en particulier que le droit à me faire assister par une personne de confiance de mon choix m'a été communiqué.

Je reconnais avoir été informé(e) du fait que l'étude pouvait être interrompue à tout moment sur décision du Promoteur ou des autorités et que toutes les mesures seraient prises dans ce cas pour assurer ma sécurité et, le cas échéant, la poursuite de mon traitement.

Je reconnais avoir été informé(e) du fait que ma participation personnelle à l'étude pouvait être suspendue si je n'en respectais pas le protocole.

J'ai bien compris que tout fait nouveau susceptible de remettre en cause mon consentement à ma participation à l'étude me serait communiqué.

Je m'engage à observer les contraintes expliquées et spécifiées dans le document d'information, à la fois pour minimiser les risques et pour la bonne réalisation du protocole.

Je m'engage notamment à ne prendre aucun médicament autre que ceux fixés par le protocole de l'étude sans l'autorisation du médecin qui dirige l'étude ou mon médecin traitant.

J'informerai le plus tôt possible le médecin de l'étude en cas de prise d'un médicament non autorisé.

J'atteste formellement ne pas être en période d'exclusion d'une autre recherche biomédicale et ne participer à aucune étude en cours.

J'ai bien compris que le fait de dissimuler la vérité peut entraîner des conséquences préjudiciables pour ma santé. J'atteste en conséquence avoir répondu de façon sincère à toutes les questions qui m'ont été posées, notamment à celles relatives à mon état de santé et à mes habitudes de vie.

Je m'engage à respecter les règles de confidentialité s'appliquant à l'étude, telles qu'elles m'ont été expliquées au préalable.

Deux exemplaires de ce consentement ont été remplis ; un exemplaire m'a été remis, l'autre étant destiné au médecin.

Fait à …………………………………………………. le ……………………………

Signature du patient

(Faire précéder de « Lu, compris et approuvé »)

Identification et coordonnées de l'Investigateur cosignataire
Signature de l'Investigateur
